# Supplementary material for: Hybrid Models and Biological Model Reduction with PyDSTool
Source: PLoS Comput Biol. 2012 Aug 9;8(8):e1002628. doi: 10.1371/journal.pcbi.1002628 (PMC3415397; doi:10.1371/journal.pcbi.1002628)
Supplement: Text S4 — Complete source code for the PyDSTool package (version 0.88.120504). Includes API documentation and help files linking to web pages. This file is identical to the current public release on Sourceforge.net. (ZIP) [file pcbi.1002628.s004.zip › PyDSTool/html/PyDSTool.Toolbox.phaseplane-pysrc.html]

xml version="1.0" encoding="ascii"?


PyDSTool.Toolbox.phaseplane


| Home | Trees | Indices | Help | PyDSTool |
| --- | --- | --- | --- | --- |

Package PyDSTool ::
Package Toolbox ::
Module phaseplane

[hide private]

[frames] | no frames]

# Source Code for Module PyDSTool.Toolbox.phaseplane

```
   1  """ 
   2      Phase plane utilities. 
   3   
   4      Some 2011 functionality has not yet been updated to use the plotter 
   5      phase plane plotting manager. 
   6   
   7   
   8  IMPORTANT NOTE DURING DEVELOPMENT: 
   9  For now, many operations with nullclines assume that they are NOT multi-valued 
  10  as a function of their variables, and that they are monotonic only as the x 
  11  variable increases. 
  12   
  13  R. Clewley, 2006 - 2011 
  14  """ 
  15   
  16  from __future__ import division 
  17  # itertools, operator used for _filter_consecutive function 
  18  import itertools, operator 
  19   
  20  from PyDSTool import * 
  21  from PyDSTool.errors import PyDSTool_ValueError 
  22  from PyDSTool.MProject import * 
  23  from PyDSTool.utils import findClosestPointIndex 
  24  from PyDSTool.common import args, metric, metric_L2, metric_weighted_L2, \ 
  25       metric_float, remain, fit_quadratic, fit_exponential, fit_diff_of_exp, \ 
  26       smooth_pts, nearest_2n_indices, make_poly_interpolated_curve, simple_bisection 
  27  from PyDSTool.common import _seq_types, _num_types 
  28  import PyDSTool.Redirector as redirc 
  29   
  30  import numpy as np 
  31  try: 
  32      from numpy import unique 
  33  except ImportError: 
  34      # older version of numpy 
  35      from numpy import unique1d as unique 
  36  import matplotlib.pyplot as pp 
  37   
  38  from scipy.interpolate import UnivariateSpline, InterpolatedUnivariateSpline 
  39  from scipy.optimize import fsolve, minpack 
  40  from scipy.optimize import minpack, zeros 
  41  try: 
  42      newton_meth = minpack.newton 
  43  except AttributeError: 
  44      # newer version of scipy 
  45      newton_meth = zeros.newton 
  46  from scipy import linspace, isfinite, sign, alltrue, sometrue, arctan 
  47   
  48  from random import uniform 
  49  import copy 
  50  import sys 
  51   
  52  norm = np.linalg.norm 
  53   
  54  # ---------------------------------------------------------------------------- 
  55   
  56  _functions = ['find_nullclines', 'make_distance_to_line_auxfn', 
  57                'make_distance_to_known_line_auxfn', 'crop_2D', 
  58                'find_period', 'make_flow_normal_event', 'filter_NaN', 
  59                'find_fixedpoints', 'find_steadystates', 'find_equilibria', 
  60                'get_perp', 'get_orthonormal', 'get_rotated', 'angle_to_vertical', 
  61                'is_min_bracket', 'find_nearest_sample_points_by_angle', 
  62                'closest_perp_distance_between_splines', 
  63                'closest_perp_distance_between_sample_points', 
  64                'closest_perp_distance_on_spline', 
  65                'line_intersection', 'find_saddle_manifolds', 
  66                'plot_PP_vf', 'plot_PP_fps', 'show_PPs', 'get_PP'] 
  67   
  68  _classes = ['distance_to_pointset', 'mesh_patch_2D', 'dx_scaled_2D', 
  69              'phaseplane', 'fixedpoint_nD', 'fixedpoint_2D', 'nullcline', 
  70              'Point2D', 'plotter_2D'] 
  71   
  72  _features = ['inflection_zone_leaf', 'inflection_zone_node', 
  73               'max_curvature_zone_leaf', 'max_curvature_zone_node'] 
  74   
  75  __all__ = _functions + _classes + _features + ['plotter'] 
  76   
  77  # ---------------------------------------------------------------------------- 
  78   
  79   


80 -class distance_to_pointset(object):


81      """First and second maximum and/or minimum distances of a point q 
  82      to a set of points, returning a dictionary keyed by 'min' and 
  83      'max' to dictionaries keyed by integers 1 and 2 (respectively). 
  84      The values of this dictionary are dictionaries of 
  85        'd' -> distance 
  86        'pos' -> index into pts 
  87   
  88      To restrict the search to a lower-dimensional subspace, specify a sub-set 
  89      of the variables in q. Defaults to Euclidean distance (normord=2),otherwise 
  90      specify metric-defining norm order. 
  91   
  92      To speed up the search, use n > 1 to create n segments of the pointset, 
  93      from which representative points will first be assessed, before the search 
  94      narrows to a particular segment. Be sure to use a large enough n given 
  95      the variation in each segment (which will otherwise cause spurious results 
  96      for certain test points). Default n=30. 
  97      [NB This option currently only returns the first max/min distances] 
  98   
  99      The radius option provides an opportunity to use information from 
 100      a previous call. This works for minimum distances only, and forces 
 101      the same segment to be searched, provided that the new point is 
 102      within the radius of the previous point (using 2-norm unless a 2D 
 103      Point is specified). CURRENTLY, THIS DOES NOT WORK WELL. KEEP IT 
 104      SWITCHED OFF USING (default) radius=0. 
 105   
 106      If gen is not None, it should contain a generator for use with isochron-related 
 107      events, for more accurate computations. The remaining arguments are associated 
 108      with this usage. 
 109      """ 
 110   
 111      _keys = ['d', 'pos'] 
 112   


113 -    def __init__(self, pts, n=30, radius=0, gen=None, iso_ev=None, other_evnames=None, 
 114                   pars_to_vars=None):


115          self.all_pts = pts 
 116          self.radius = radius 
 117          num_pts = len(pts) 
 118          assert n < num_pts, "Use a larger pointset or fewer segments" 
 119          assert n > 0, "n must be a positive integer" 
 120          assert type(n) == int, "n must be a positive integer" 
 121          self.gen = gen 
 122          self.iso_ev = iso_ev 
 123          self.other_evnames = other_evnames 
 124          self.pars_to_vars = pars_to_vars 
 125          if n == 1: 
 126              # always search whole pointset 
 127              self.byseg = False 
 128              self.segments = None 
 129              self.rep_pts = None 
 130              self.seg_start_ixs = None 
 131          else: 
 132              # use segments to speed up search 
 133              self.byseg = True 
 134              step, m = divmod(num_pts, n) 
 135              if step < 2: 
 136                  # Not enough points to use segments 
 137                  self.byseg = False 
 138                  self.segments = None 
 139                  self.rep_pts = None 
 140                  self.seg_start_ixs = None 
 141              else: 
 142                  half_step = int(floor(step/2.)) 
 143                  # space the segment representatives in middle of segments 
 144                  self.seg_start_ixs = [step*i for i in range(n)] 
 145                  rep_pts = [pts[step*i+half_step] for i in range(n)] 
 146                  # treat any remainder segment specially 
 147                  # if remainder is too small then combine it with previous 
 148                  # segment (choose m>=6 fairly arbitrarily) 
 149                  self.segments = [] 
 150                  base_ix = 0 
 151                  for i in range(n): 
 152                      self.segments.append(pts[base_ix:base_ix+step]) 
 153                      base_ix += step 
 154                  if m >= 6: 
 155                      half_step_last = int(floor(m/2.)) 
 156                      self.segments.append(pts[base_ix:]) 
 157                      rep_pts.append(pts[n*half_step+half_step_last]) 
 158                      self.seg_start_ixs.append(step*n) 
 159                  else: 
 160                      # replace last segment with all the rest of the points, 
 161                      # making it longer 
 162                      self.segments[-1] = pts[base_ix-step:] 
 163                      # leave rep_pts[-1] alone 
 164                  self.rep_pts = pointsToPointset(rep_pts) 
 165          # record of [last segment identified for 1st and 2nd MINIMUM distance, 
 166          #                                                           last point] 
 167          # max distances are not currently recorded 
 168          self.clear_history()

 169   


170 -    def clear_history(self):


171          self.history = [None, None, None]

 172   


173 -    def __call__(self, q, use_norm=False, normord=2, minmax=['min', 'max']):


174          dic_info = self._distance(q, use_norm, normord, minmax) 
 175          if self.gen is not None: 
 176              # refine distance using gen_test_fn 
 177              perp_ev, t_ev = _find_min_pt(self.gen, q, 
 178                                           dic_info['min'][1]['pos'], self.all_pts, 
 179                                           self.pars_to_vars, self.iso_ev, 
 180                                           self.other_evnames) 
 181              ev_pt = perp_ev[0][q.coordnames] 
 182              if use_norm: 
 183                  dic_info['min'][0] = norm(q - ev_pt, normord) 
 184              else: 
 185                  m = q - ev_pt 
 186                  try: 
 187                      vars = q.coordnames 
 188                  except AttributeError: 
 189                      raise TypeError("Input point must be a Point object") 
 190                  m[vars[0]] = abs(m[vars[0]]) 
 191                  m[vars[1]] = abs(m[vars[1]]) 
 192                  dic_info['min'][0] = m 
 193              # leave pos information alone -- it says what the nearest point is in 
 194              # self.all_pts 
 195          return dic_info

 196   


197 -    def _distance(self, q, use_norm, normord, minmax):


198          try: 
 199              vars = q.coordnames 
 200          except AttributeError: 
 201              raise TypeError("Input point must be a Point object") 
 202          # HACK: cannot use Inf in dmin for these comparisons 
 203          # have to use a big number 
 204          if use_norm: 
 205              if 'min' in minmax: 
 206                  dmin = (_num_inf, NaN) 
 207                  dmin_old = (_num_inf, NaN) 
 208                  def le(a,b): 
 209                      return a < b

 210              if 'max' in minmax: 
 211                  dmax = (0, NaN) 
 212                  dmax_old = (0, NaN) 
 213                  def ge(a,b): 
 214                      return a > b

 215              def fn(p,q): 
 216                  return norm(p-q, normord) 
 217          else: 
 218              if 'min' in minmax: 
 219                  dmin = (array([_num_inf for v in vars]), NaN) 
 220                  dmin_old = (array([_num_inf for v in vars]), NaN) 
 221                  def le(a,b): 
 222                      return sometrue(a-b<0) 
 223              if 'max' in minmax: 
 224                  dmax = (array([0. for v in vars]), NaN) 
 225                  dmax_old = (array([0. for v in vars]), NaN) 
 226                  def ge(a,b): 
 227                      return sometrue(a-b>0) 
 228              def fn(p,q): 
 229                  m = p-q 
 230                  m[vars[0]] = abs(m[vars[0]]) 
 231                  m[vars[1]] = abs(m[vars[1]]) 
 232                  return m 
 233  #        if remain(self.all_pts.coordnames, vars) != []: 
 234  #            raise TypeError("Input points must be a Pointset object sharing " 
 235  #                            "common coordinate names") 
 236          if self.byseg: 
 237              if self.history[0] is None or self.radius == 0: 
 238                  use_history = False 
 239              else: 
 240                  # use last known segment only if q is within radius of 
 241                  # last q 
 242                  try: 
 243                      test = q-self.history[2]   # a Point 
 244                  except: 
 245                      # problem with self.history[2] (externally tampered with?) 
 246                      print "History is: ", self.history 
 247                      raise RuntimeError("Invalid history object in " 
 248                                         "distance_to_pointset class instance") 
 249                  try: 
 250                      use_history = abs(test[0]) < self.radius[0] and \ 
 251                         abs(test[1]) < self.radius[1] 
 252                  except: 
 253                      # radius is a scalar 
 254                      use_history = norm(test) < self.radius 
 255              if use_history and minmax == ['min']: 
 256                      res_min1 = self.search_min(q, self.segments[self.history[0]][vars], 
 257                                      fn, le, dmin, dmin_old) 
 258                      res_min2 = self.search_min(q, self.segments[self.history[1]][vars], 
 259                                      fn, le, dmin, dmin_old) 
 260                      rm1 = res_min1['min'][1] 
 261                      rm2 = res_min2['min'][1] 
 262                      if rm1['d'] < rm2['d']: 
 263                          seg_min_pos1 = rm1['pos'] 
 264                          seg_min_d1 = rm1['d'] 
 265                          seg_min1 = self.history[0] 
 266                      else: 
 267                          seg_min_pos1 = rm2['pos'] 
 268                          seg_min_d1 = rm2['d'] 
 269                          seg_min1 = self.history[1] 
 270                      global_min_ix1 = self.seg_start_ixs[seg_min1] + seg_min_pos1 
 271                      return {'min': {1: {'d': seg_min_d1, 'pos': global_min_ix1}, 
 272                                      2: {'d': None, 'pos': None}}} 
 273              else: 
 274                  if 'min' in minmax and 'max' in minmax: 
 275                      first_pass = self.search_both(q, self.rep_pts[vars], 
 276                                   fn, le, ge, dmin, dmin_old, dmax, dmax_old) 
 277                      # search 1st and 2nd segments for min and max 
 278                      # the 'pos' index will be into the self.segments list 
 279                      seg_ixs_min = [first_pass['min'][1]['pos']] 
 280                      seg_ixs_min.append(first_pass['min'][2]['pos']) 
 281                      seg_ixs_max = [first_pass['max'][1]['pos']] 
 282                      seg_ixs_max.append(first_pass['max'][2]['pos']) 
 283                      # min 
 284                      res_min1 = self.search_min(q, self.segments[seg_ixs_min[0]][vars], 
 285                                      fn, le, dmin, dmin_old) 
 286                      res_min2 = self.search_min(q, self.segments[seg_ixs_min[1]][vars], 
 287                                      fn, le, dmin, dmin_old) 
 288                      rm1 = res_min1['min'][1] 
 289                      rm2 = res_min2['min'][1] 
 290                      if rm1['d'] < rm2['d']: 
 291                          seg_min_pos1 = rm1['pos'] 
 292                          seg_min_d1 = rm1['d'] 
 293                          seg_min1 = seg_ixs_min[0] 
 294                          seg_min2 = seg_ixs_min[1] 
 295                      else: 
 296                          seg_min_pos1 = rm2['pos'] 
 297                          seg_min_d1 = rm2['d'] 
 298                          seg_min1 = seg_ixs_min[1] 
 299                          seg_min2 = seg_ixs_min[0] 
 300                      global_min_ix1 = self.seg_start_ixs[seg_min1] + seg_min_pos1 
 301                      # do second minimum here (NOT IMPLEMENTED) 
 302                      # max 
 303                      res_max1 = self.search_max(q, self.segments[seg_ixs_max[0]][vars], 
 304                                      fn, ge, dmax, dmax_old) 
 305                      res_max2 = self.search_max(q, self.segments[seg_ixs_max[1]][vars], 
 306                                      fn, ge, dmax, dmax_old) 
 307                      rm1 = res_max1['max'][1] 
 308                      rm2 = res_max2['max'][1] 
 309                      if rm1['d'] < rm2['d']: 
 310                          seg_max_pos1 = rm1['pos'] 
 311                          seg_max_d1 = rm1['d'] 
 312                          seg_max1 = seg_ixs_max[0] 
 313                      else: 
 314                          seg_max_pos1 = rm2['pos'] 
 315                          seg_max_d1 = rm2['d'] 
 316                          seg_max1 = seg_ixs_max[1] 
 317                      global_max_ix1 = self.seg_start_ixs[seg_max1] + seg_max_pos1 
 318                      # do second maximum here (NOT IMPLEMENTED) 
 319                      # array(q) is a cheap way to take a copy of q without 
 320                      # making a new Point object 
 321                      self.history = [seg_min1, seg_min2, array(q)] 
 322                      return {'min': {1: {'d': seg_min_d1, 'pos': global_min_ix1}, 
 323                                      2: {'d': None, 'pos': None}}, 
 324                              'max': {1: {'d': seg_max_d1, 'pos': global_max_ix1}, 
 325                                      2: {'d': None, 'pos': None}}} 
 326                  elif 'min' in minmax: 
 327                      first_pass = self.search_min(q, self.rep_pts[vars], 
 328                                   fn, le, dmin, dmin_old) 
 329                      seg_ixs_min = [first_pass['min'][1]['pos']] 
 330                      seg_ixs_min.append(first_pass['min'][2]['pos']) 
 331                      res_min1 = self.search_min(q, self.segments[seg_ixs_min[0]][vars], 
 332                                      fn, le, dmin, dmin_old) 
 333                      res_min2 = self.search_min(q, self.segments[seg_ixs_min[1]][vars], 
 334                                      fn, le, dmin, dmin_old) 
 335                      rm1 = res_min1['min'][1] 
 336                      rm2 = res_min2['min'][1] 
 337                      if rm1['d'] < rm2['d']: 
 338                          seg_min_pos1 = rm1['pos'] 
 339                          seg_min_d1 = rm1['d'] 
 340                          seg_min1 = seg_ixs_min[0] 
 341                          seg_min2 = seg_ixs_min[1] 
 342                      else: 
 343                          seg_min_pos1 = rm2['pos'] 
 344                          seg_min_d1 = rm2['d'] 
 345                          seg_min1 = seg_ixs_min[1] 
 346                          seg_min2 = seg_ixs_min[0] 
 347                      global_min_ix1 = self.seg_start_ixs[seg_min1] + seg_min_pos1 
 348                      # do second minimum here (NOT IMPLEMENTED) 
 349                      # array(q) is a cheap way to take a copy of q without 
 350                      # making a new Point object 
 351                      self.history = [seg_min1, seg_min2, array(q)] 
 352                      return {'min': {1: {'d': seg_min_d1, 'pos': global_min_ix1}, 
 353                                      2: {'d': None, 'pos': None}}} 
 354                  elif 'max' in minmax: 
 355                      first_pass = self.search_max(q, self.rep_pts[vars], 
 356                                   fn, ge, dmax, dmax_old) 
 357                      seg_ixs_max = [first_pass['max'][1]['pos']] 
 358                      seg_ixs_max.append(first_pass['max'][2]['pos']) 
 359                      res_max1 = self.search_max(q, self.segments[seg_ixs_max[0]][vars], 
 360                                      fn, ge, dmax, dmax_old) 
 361                      res_max2 = self.search_max(q, self.segments[seg_ixs_max[1]][vars], 
 362                                      fn, ge, dmax, dmax_old) 
 363                      rm1 = res_max1['max'][1] 
 364                      rm2 = res_max2['max'][1] 
 365                      if rm1['d'] < rm2['d']: 
 366                          seg_max_pos1 = rm1['pos'] 
 367                          seg_max_d1 = rm1['d'] 
 368                          seg_max1 = seg_ixs_max[0] 
 369                      else: 
 370                          seg_max_pos1 = rm2['pos'] 
 371                          seg_max_d1 = rm2['d'] 
 372                          seg_max1 = seg_ixs_max[1] 
 373                      global_max_ix1 = self.seg_start_ixs[seg_max1] + seg_max_pos1 
 374                      # do second maximum here (NOT IMPLEMENTED) 
 375                      # don't update history because it's only for minimum distance 
 376                      return {'max': {1: {'d': seg_max_d1, 'pos': global_max_ix1}, 
 377                                      2: {'d': None, 'pos': None}}} 
 378                  else: 
 379                      raise RuntimeError("Invalid min/max option") 
 380          else: 
 381              if 'min' in minmax and 'max' in minmax: 
 382                  return self.search_both(q, self.all_pts[vars], 
 383                               fn, le, ge, dmin, dmin_old, dmax, dmax_old) 
 384              elif 'min' in minmax: 
 385                  return self.search_min(q, self.all_pts[vars], 
 386                               fn, le, dmin, dmin_old) 
 387              elif 'max' in minmax: 
 388                  return self.search_max(q, self.all_pts[vars], 
 389                               fn, ge, dmax, dmax_old) 
 390              else: 
 391                  raise RuntimeError("Invalid min/max option") 
 392   
 393   


394 -    def search_both(self, q, pts, 
 395                 fn, le, ge, dmin, dmin_old, dmax, dmax_old):


396          for i, p in enumerate(pts): 
 397              d = fn(p,q) 
 398              if le(d, dmin[0]): 
 399                  dmin_old = dmin 
 400                  dmin = (d, i) 
 401              elif le(d, dmin_old[0]): 
 402                  dmin_old = (d, i) 
 403              if ge(d, dmax[0]): 
 404                  dmax_old = dmax 
 405                  dmax = (d, i) 
 406              elif ge(d, dmax_old[0]): 
 407                  dmax_old = (d,i) 
 408          return {'min': {1: dict(zip(self._keys,dmin)), 
 409                          2: dict(zip(self._keys,dmin_old))}, 
 410                  'max': {1: dict(zip(self._keys,dmax)), 
 411                          2: dict(zip(self._keys,dmax_old))}}

 412   


413 -    def search_min(self, q, pts, fn, le, dmin, dmin_old):


414          for i, p in enumerate(pts): 
 415              d = fn(p,q) 
 416              if le(d, dmin[0]): 
 417                  dmin_old = dmin 
 418                  dmin = (d, i) 
 419              elif le(d, dmin_old[0]): 
 420                  dmin_old = (d, i) 
 421          return {'min': {1: dict(zip(self._keys,dmin)), 
 422                          2: dict(zip(self._keys,dmin_old))}}

 423   


424 -    def search_max(self, q, pts, fn, ge, dmax, dmax_old):


425          for i, p in enumerate(pts): 
 426              d = fn(p,q) 
 427              if ge(d, dmax[0]): 
 428                  dmax_old = dmax 
 429                  dmax = (d, i) 
 430              elif ge(d, dmax_old[0]): 
 431                  dmax_old = (d,i) 
 432          return {'max': {1: dict(zip(self._keys,dmax)), 
 433                          2: dict(zip(self._keys,dmax_old))}}

 434   
 435   


436 -def find_nullclines(gen, xname, yname, subdomain=None, fps=None, n=10, 
 437                      t=0, eps=None, crop_tol_pc=0.01, jac=None, max_step=0, 
 438                      max_num_points=1000, only_var=None, seed_points=None, 
 439                      strict_domains=True, pycont_cache=None):


440      """Find nullclines of a two-dimensional sub-system of the given 
 441      Generator object gen, specified by xname and yname. 
 442   
 443      Inputs: 
 444   
 445      gen is a Generator object 
 446      xname, yname are state variable names of gen 
 447   
 448      Optional inputs: 
 449   
 450      Restriction of x and y to sub-domains can be made using the subdomain argument. 
 451      subdomain may contain pairs (min, max) or singleton values that fix those state 
 452      variables and restrict the fixed point search to the remaining sub-system on the 
 453      given ranges. (default to domains given in generator). There must be exactly 
 454      two ranges remaining (for xname, yname) to give a two-dimensional nullcline 
 455      problem, unless only_var option has been used (see below). 
 456   
 457      n = initial number of meshpoints for fsolve. Do not set this large if using 
 458        PyCont, e.g. use n=3. Default is 10. 
 459   
 460      Set t value for non-autonomous systems (default 0). Support for Jacobians 
 461        with non-autonomous systems is not yet provided. 
 462   
 463      jac is a Jacobian function that accepts keyword arguments including t for 
 464        time (even if Jacobian is time-independent). 
 465   
 466      max_step (dictionary) tells PyCont the largest step size to use for each 
 467        variable. Integer 0 (default) switches off PyCont use. Use None 
 468        to tell PyCont to use default max step size (5e-1). 
 469   
 470      fps can be a list of points previously calculated to be fixed points, 
 471        which this function will use as additional starting points for 
 472        computation. 
 473   
 474      eps sets the accuracy to which the nullclines are calculated. Default is 
 475        approximately 1e-8. 
 476   
 477      crop_tol_pc is the percentage (default 1%) for tolerance outside the specified 
 478        domain for keeping points in nullclines -- final points will be cropped with 
 479        this tolerance. 
 480   
 481      only_var (variable name) requests that only the nullcline for that variable 
 482        will be computed. The variable name must correspond to xname or yname. 
 483   
 484      seed_points can be used if an approximate position on either nullcline is already 
 485        known, especially useful if there are no fixed points provided. One or more 
 486        seed points should be given as a list of dictionaries or Points with keys or 
 487        coordinates xname and yname. These should be collected as values of the 
 488        seed_points dictionary, whose keys (xname and/or yname) indicate which points 
 489        belong to which nullclines. 
 490   
 491      strict_domains (boolean, default True) ensures all computed nullcline points are 
 492        cropped to lie within the given sub-domains. 
 493   
 494      pycont_cache (list of two PyCont objects, default None) can be provided to 
 495        improve efficiency of PyCont use, to avoid re-creation of ODE objects for 
 496        each call to this function. If a list of [None, None] is provided, this will 
 497        ensure that a list of the two PyCont objects will be returned in an additional 
 498        output. !!Don't use this cache if external inputs to the Generator have changed!! 
 499   
 500      Output: 
 501          (x_null, y_null) arrays of (x,y) pairs. Fixed points will be included if 
 502          they are provided (they are expected to be calculated to high enough 
 503          accuracy to smoothly fit with the nullcline sample points). 
 504   
 505          (optional) [P_x, P_y] list of PyCont objects, returned if 
 506            pycont_cache input option is used (see above). 
 507   
 508      Note that the points returned are not guaranteed to be in any particular 
 509      order when PyCont is not used. 
 510      """ 
 511      vardict = filteredDict(copy.copy(gen.initialconditions), gen.funcspec.vars) 
 512      # get state variable domains if subdomain dictionary not given 
 513      if subdomain is None: 
 514          subdomain = filteredDict(gen.xdomain, gen.funcspec.vars) 
 515      else: 
 516          subdomain = gen._FScompatibleNames(subdomain) 
 517          assert remain(subdomain.keys(),gen.funcspec.vars) == [] and \ 
 518                 remain(gen.funcspec.vars,subdomain.keys()) == [] 
 519      if only_var is None: 
 520          do_vars = [xname, yname] 
 521      else: 
 522          assert only_var in [xname, yname], "only_var must be one of xname or yname" 
 523          do_vars = [only_var] 
 524      fixed_vars = {} 
 525      try: 
 526          x_dom = subdomain[xname] 
 527      except: 
 528          x_dom = gen.xdomain[xname] 
 529      if not (isfinite(x_dom[0]) and isfinite(x_dom[1])): 
 530          raise PyDSTool_ExistError("Must specify finite range for %s"%xname) 
 531      try: 
 532          y_dom = subdomain[yname] 
 533      except: 
 534          y_dom = gen.xdomain[yname] 
 535      if not (isfinite(y_dom[0]) and isfinite(y_dom[1])): 
 536          raise PyDSTool_ExistError("Must specify finite range for %s"%yname) 
 537      for varname, dom in subdomain.iteritems(): 
 538          if isinstance(dom, (tuple,list)): 
 539              if not (isfinite(dom[0]) and isfinite(dom[1])): 
 540                  raise RuntimeError("Must specify a finite range for %s" % varname) 
 541          else: 
 542              fixed_vars[varname] = dom 
 543      if fixed_vars != {}: 
 544          vardict.update(filteredDict(gen._FScompatibleNames(dict(fixed_vars)), 
 545                          remain(gen.funcspec.vars, [xname, yname]))) 
 546      var_ix_map = invertMap(gen.funcspec.vars) 
 547      max_step = gen._FScompatibleNames(max_step) 
 548      xname_orig = gen._FScompatibleNamesInv(xname) 
 549      yname_orig = gen._FScompatibleNamesInv(yname) 
 550      xname = gen._FScompatibleNames(xname) 
 551      yname = gen._FScompatibleNames(yname) 
 552      x_ix = var_ix_map[xname] 
 553      y_ix = var_ix_map[yname] 
 554      pp_vars = [xname, yname] 
 555      pp_vars.sort() 
 556      pp_var_ix_map = invertMap(pp_vars) 
 557   
 558      # subscript refers to the component returned 
 559      # first letter refers to order of args 
 560      def xdot_x(x, y, t): 
 561          vardict[yname] = y 
 562          vardict[xname] = x 
 563          try: 
 564              return gen.Rhs(t,vardict,gen.pars)[x_ix] 
 565          except OverflowError: 
 566              pass

 567      def ydot_y(y, x, t): 
 568          vardict[yname] = y 
 569          vardict[xname] = x 
 570          try: 
 571              return gen.Rhs(t,vardict,gen.pars)[y_ix] 
 572          except OverflowError: 
 573              pass 
 574      if gen.haveJacobian(): 
 575          # user-supplied Jacobian if present 
 576          # subscript refers to the component returned 
 577          def xfprime_x(x, y, t): 
 578              vardict[xname] = x 
 579              vardict[yname] = y 
 580              try: 
 581                  return gen.Jacobian(t, vardict, gen.pars)[x_ix] 
 582              except OverflowError: 
 583                  pass 
 584          def yfprime_y(y, x, t): 
 585              vardict[xname] = x 
 586              vardict[yname] = y 
 587              try: 
 588                  return gen.Jacobian(t, vardict, gen.pars)[y_ix] 
 589              except OverflowError: 
 590                  pass 
 591      elif jac is not None: 
 592          # assumes **args-compatible signature! 
 593          # subscript refers to the component returned 
 594          xix = pp_var_ix_map[xname] 
 595          yix = pp_var_ix_map[yname] 
 596          xfprime_x = lambda x, y, t: array(jac(**{'t': t, xname: float(x), 
 597                                        yname: float(y)})[xix]) 
 598          yfprime_y = lambda y, x, t: array(jac(**{'t': t, xname: float(x), 
 599                                        yname: float(y)})[yix]) 
 600      else: 
 601          xfprime_x = None 
 602          yfprime_y = None 
 603   
 604      if eps is None: 
 605          eps = 1.49012e-8 
 606   
 607      # ranges for initial search for nullcline points 
 608      x_range = list(linspace(gen.xdomain[xname][0],gen.xdomain[xname][1],n)) 
 609      y_range = list(linspace(gen.xdomain[yname][0],gen.xdomain[yname][1],n)) 
 610   
 611      if fps is not None: 
 612          fps_FS = [gen._FScompatibleNames(fp) for fp in fps] 
 613          add_pts_x = [fp[xname] for fp in fps_FS] 
 614          add_pts_y = [fp[yname] for fp in fps_FS] 
 615      else: 
 616          add_pts_x = [] 
 617          add_pts_y = [] 
 618   
 619      x_null_pts = [] 
 620      y_null_pts = [] 
 621   
 622      seed_pts_x = [] 
 623      seed_pts_y = [] 
 624      if x_dom != gen.xdomain[xname] or y_dom != gen.xdomain[yname]: 
 625          # user sub-domains - make sure there's at least one start point inside 
 626          seed_pts_x.append( 0.5*(x_dom[0]+x_dom[1]) ) 
 627          seed_pts_y.append( 0.5*(y_dom[0]+y_dom[1]) ) 
 628   
 629      if seed_points is not None: 
 630          seed_points = gen._FScompatibleNames(seed_points) 
 631          if yname in seed_points: 
 632              seed_pts_x_for_ynull = [pt[xname] for pt in seed_points[yname]] 
 633              seed_pts_y_for_ynull = [pt[yname] for pt in seed_points[yname]] 
 634          else: 
 635              seed_pts_x_for_ynull = [] 
 636              seed_pts_y_for_ynull = [] 
 637          if xname in seed_points: 
 638              seed_pts_x_for_xnull = [pt[xname] for pt in seed_points[xname]] 
 639              seed_pts_y_for_xnull = [pt[yname] for pt in seed_points[xname]] 
 640          else: 
 641              seed_pts_x_for_xnull = [] 
 642              seed_pts_y_for_xnull = [] 
 643          seed_pts_x.extend(seed_pts_x_for_xnull+seed_pts_x_for_ynull) 
 644          seed_pts_y.extend(seed_pts_y_for_xnull+seed_pts_y_for_ynull) 
 645      else: 
 646          seed_pts_x_for_ynull = seed_pts_x_for_xnull = [] 
 647          seed_pts_y_for_ynull = seed_pts_y_for_xnull = [] 
 648   
 649      x_range = seed_pts_x + add_pts_x + x_range 
 650      y_range = seed_pts_y + add_pts_y + y_range 
 651   
 652      # try to improve seed points or otherwise use crappy fsolve to find some 
 653      # points on the nullclines. 
 654      rout = redirc.Redirector(redirc.STDOUT) 
 655      rout.start() 
 656      if yname in do_vars: 
 657          for x0 in x_range: 
 658              try: 
 659                  x_null_pts.extend([(_xinf_ND(xdot_x,x0,args=(y,t), 
 660                                 xddot=xfprime_x,xtol=eps/10.),y) for y in y_range]) 
 661                  y_null_pts.extend([(x0,_xinf_ND(ydot_y,y,args=(x0,t), 
 662                                 xddot=yfprime_y,xtol=eps/10.)) for y in y_range]) 
 663              except (OverflowError, ZeroDivisionError): 
 664                  rout.stop() 
 665              except: 
 666                  rout.stop() 
 667                  raise 
 668      if xname in do_vars: 
 669          for y0 in y_range: 
 670              try: 
 671                  x_null_pts.extend([(_xinf_ND(xdot_x,x,args=(y0,t), 
 672                                 xddot=xfprime_x,xtol=eps/10.),y0) for x in x_range]) 
 673                  y_null_pts.extend([(x,_xinf_ND(ydot_y,y0,args=(x,t), 
 674                                 xddot=yfprime_y,xtol=eps/10.)) for x in x_range]) 
 675              except (OverflowError, ZeroDivisionError): 
 676                  rout.stop() 
 677              except: 
 678                  rout.stop() 
 679                  raise 
 680      rout.stop() 
 681   
 682      # intervals based on user specs (if different to inherent variable domains) 
 683      xwidth = abs(x_dom[1]-x_dom[0]) 
 684      ywidth = abs(y_dom[1]-y_dom[0]) 
 685      xinterval=Interval('xdom', float, [x_dom[0]-crop_tol_pc*xwidth, 
 686                                         x_dom[1]+crop_tol_pc*xwidth]) 
 687      yinterval=Interval('ydom', float, [y_dom[0]-crop_tol_pc*ywidth, 
 688                                         y_dom[1]+crop_tol_pc*ywidth]) 
 689   
 690      x_null_fsolve = filter_close_points(crop_2D(filter_NaN(x_null_pts), 
 691                                  xinterval, yinterval), eps*10) 
 692      y_null_fsolve = filter_close_points(crop_2D(filter_NaN(y_null_pts), 
 693                                  xinterval, yinterval), eps*10) 
 694   
 695      # intervals based on inherent variable domains 
 696      if x_dom == gen.xdomain[xname]: 
 697          x_null_inh = [] 
 698          y_null_inh = [] 
 699      else: 
 700          x_dom_inh = gen.xdomain[xname] 
 701          y_dom_inh = gen.xdomain[yname] 
 702          xinterval_inh = Interval('xdom', float, [x_dom_inh[0], x_dom_inh[1]]) 
 703          yinterval_inh = Interval('ydom', float, [y_dom_inh[0], y_dom_inh[1]]) 
 704          x_null_inh = filter_close_points(crop_2D(filter_NaN(x_null_pts), 
 705                                  xinterval_inh, yinterval_inh), eps*10) 
 706          x_null_inh.sort(1) 
 707          y_null_inh = filter_close_points(crop_2D(filter_NaN(y_null_pts), 
 708                                  xinterval_inh, yinterval_inh), eps*10) 
 709          y_null_inh.sort(0) 
 710   
 711   
 712      # default value for now 
 713      do_cache = False 
 714      # max_step = 0 means do not use PyCont to improve accuracy 
 715      if max_step != 0: 
 716          if pycont_cache is None: 
 717              pycont_cache = [None, None]  # dummy values 
 718              do_cache = False 
 719          else: 
 720              do_cache = True 
 721          add_fp_pts = array([add_pts_x, add_pts_y]).T 
 722          if not isinstance(max_step, dict): 
 723              max_step = {xname: max_step, yname: max_step} 
 724          # use PyCont to improve accuracy 
 725          # PyCont will order the points so can draw lines 
 726          loop_step = 5 
 727          # current limitation! 
 728          # pycont won't find disconnected branches of nullclines so we have to seed 
 729          # with multiple starting points 
 730          # - also, the following assumes more than one point was already added to 
 731          # these lists 
 732   
 733          # Y 
 734          if yname in do_vars: 
 735              # set up a prioritized list of starting points 
 736              x_init_priority_list = [] 
 737              y_init_priority_list = [] 
 738              if fps is not None and len(fps) > 0: 
 739                  x_init = fps[0][xname_orig] + 1e-4*(x_dom[1]-x_dom[0]) 
 740                  y_init = fps[0][yname_orig] + 1e-4*(y_dom[1]-y_dom[0]) 
 741                  x_init_priority_list.append(x_init) 
 742                  y_init_priority_list.append(y_init) 
 743              if len(y_null_fsolve) > 0: 
 744                  # get an initial point from user-specified sub-domain 
 745                  index = int(len(y_null_fsolve)/2.) 
 746                  x_init, y_init = y_null_fsolve[index] 
 747                  x_init_priority_list.append(x_init) 
 748                  y_init_priority_list.append(y_init) 
 749              x_init_priority_list.extend( seed_pts_x_for_ynull ) 
 750              y_init_priority_list.extend( seed_pts_y_for_ynull ) 
 751              if len(y_null_inh) > 0: 
 752                  old_indices = [] 
 753                  for ratio in (0.25, 0.5, 0.75): 
 754                      index = int(len(y_null_inh)*ratio) 
 755                      if index in old_indices: 
 756                          continue 
 757                      else: 
 758                          old_indices.append(index) 
 759                      x_init, y_init = y_null_inh[index] 
 760                      x_init_priority_list.append(x_init) 
 761                      y_init_priority_list.append(y_init) 
 762              # lowest priority: domain midpoint 
 763              x_init_priority_list.append( (x_dom[0]+x_dom[1])/2. ) 
 764              y_init_priority_list.append( (y_dom[0]+y_dom[1])/2. ) 
 765   
 766              # initialize starting point 
 767              x_init = x_init_priority_list[0] 
 768              y_init = y_init_priority_list[0] 
 769   
 770              max_tries = min(len(x_init_priority_list), 4) 
 771   
 772              if pycont_cache[1] is None: 
 773                  sysargs_y = args(name='nulls_y', pars={xname: x_init}, 
 774                             ics={yname: y_init}, 
 775                             xdomain={yname: gen.xdomain[yname]}, pdomain={xname: gen.xdomain[xname]}) 
 776                  if 'inputs' in gen.funcspec._initargs: # and isinstance(gen.funcspec._initargs['inputs'], dict): 
 777                      if len(gen.funcspec._initargs['inputs']) > 0: 
 778                          sysargs_y['inputs'] = gen.inputs.copy() 
 779                  sysargs_y.pars.update(gen.pars) 
 780                  sysargs_y.pars.update(filteredDict(vardict, [xname, yname], neg=True)) 
 781                  varspecs = {yname: gen.funcspec._initargs['varspecs'][yname]} 
 782                  if 'fnspecs' in gen.funcspec._initargs: 
 783                      old_fnspecs = gen.funcspec._initargs['fnspecs'] 
 784                      fnspecs, new_varspecs = resolveClashingAuxFnPars(old_fnspecs, varspecs, 
 785                                                                       sysargs_y.pars.keys()) 
 786                      # TEMP 
 787                      # process any Jacobian functions to remove un-needed terms 
 788                      if 'Jacobian' in fnspecs: 
 789                          del fnspecs['Jacobian'] 
 790  ##                        fnspecs['Jacobian'] = makePartialJac(fnspecs['Jacobian'], [yname]) 
 791  ##                    if 'Jacobian_pars' in fnspecs: 
 792  ##                        fnspecs['Jacobian_pars'] = makePartialJac(fnspecs['Jacobian_pars'], [yname]) 
 793  ##                    elif 'Jacobian' in fnspecs: 
 794  ##                        old_fargs, J_par_spec = makePartialJac(old_fnspecs['Jacobian'], [yname], [xname]) 
 795  ##                        fnspecs['Jacobian_pars'] = (fnspecs['Jacobian'][0], J_par_spec) 
 796                      sysargs_y.update({'fnspecs': fnspecs}) 
 797                  else: 
 798                      new_varspecs = varspecs 
 799                  sysargs_y.varspecs = new_varspecs 
 800                  sysargs_y.pars['time'] = t 
 801                  sys_y = Vode_ODEsystem(sysargs_y) 
 802                  P_y = ContClass(sys_y) 
 803                  pycont_cache[1] = P_y 
 804              else: 
 805                  P_y = pycont_cache[1] 
 806                  new_pars = gen.pars.copy() 
 807                  new_pars.update(filteredDict(vardict, [xname, yname], neg=True)) 
 808                  new_pars['time'] = t 
 809                  P_y.model.set(pars=new_pars) 
 810              PCargs = args(name='null_curve_y', type='EP-C', force=True) 
 811              PCargs.freepars = [xname] 
 812              PCargs.MinStepSize = 1e-8 
 813              PCargs.VarTol = PCargs.FuncTol = eps*10 
 814              PCargs.TestTol = eps 
 815              if max_step is None: 
 816                  PCargs.MaxStepSize = 5e-1 
 817                  PCargs.StepSize = 1e-6 
 818              else: 
 819                  PCargs.MaxStepSize = max_step[xname] 
 820                  PCargs.StepSize = max_step[xname]*0.5 
 821              PCargs.MaxNumPoints = loop_step 
 822              PCargs.MaxCorrIters = 8 
 823              #PCargs.verbosity = 100 
 824              P_y.newCurve(PCargs) 
 825   
 826              # Continue, checking every loop_step points until go out of bounds 
 827              # FORWARD ########### 
 828              done = False 
 829              num_points = 0 
 830              in_subdom = x_init in xinterval and y_init in yinterval 
 831              tries = 0 
 832              while not done: 
 833                  try: 
 834                      P_y['null_curve_y'].forward() 
 835                  except PyDSTool_ExistError: 
 836                      tries += 1 
 837                      if tries == max_tries: 
 838                          print 'null_curve_y failed in forward direction' 
 839                          raise 
 840                      else: 
 841                          # try a different starting point 
 842                          x_init = x_init_priority_list[tries] 
 843                          y_init = y_init_priority_list[tries] 
 844                          sys_y.set(pars={xname: x_init}, ics={yname: y_init}) 
 845                  else: 
 846                      num_points += loop_step 
 847                      # stop if have tried going forwards too much and solution becomes 
 848                      # outside of sub-domains, or if num_points exceeds max_num_points 
 849                      if in_subdom: 
 850                          done = (num_points > 5*loop_step or num_points > max_num_points) 
 851                          done = done and not \ 
 852                              check_bounds(array([P_y['null_curve_y'].new_sol_segment[xname], 
 853                                                  P_y['null_curve_y'].new_sol_segment[yname]]).T, 
 854                                           xinterval, yinterval) 
 855                          y_null_part = crop_2D(array([P_y['null_curve_y'].sol[xname], 
 856                                                       P_y['null_curve_y'].sol[yname]]).T, 
 857                                                xinterval, yinterval) 
 858                      else: 
 859                          # did not start in sub-domain and still not yet found 
 860                          # it during continuation 
 861                          y_null_part = crop_2D(array([P_y['null_curve_y'].sol[xname], 
 862                                                       P_y['null_curve_y'].sol[yname]]).T, 
 863                                                xinterval, yinterval) 
 864                          in_subom = len(y_null_part)>0 
 865                          done = num_points > 15*loop_step 
 866   
 867              # BACKWARD ########### 
 868              done = False 
 869              num_points = 0 
 870              in_subdom = x_init in xinterval and y_init in yinterval 
 871              tries = 0 
 872              while not done: 
 873                  try: 
 874                      P_y['null_curve_y'].backward() 
 875                  except PyDSTool_ExistError: 
 876                      tries += 1 
 877                      if tries == max_tries: 
 878                          print 'null_curve_y failed in backward direction' 
 879                          raise 
 880                      else: 
 881                          # try a different starting point 
 882                          x_init = x_init_priority_list[tries] 
 883                          y_init = y_init_priority_list[tries] 
 884                          sys_y.set(pars={xname: x_init}, ics={yname: y_init}) 
 885                  else: 
 886                      num_points += loop_step 
 887                      # stop if have tried going backwards too much and solution becomes 
 888                      # outside of sub-domain, or if num_points exceeds max_num_points 
 889                      if in_subdom: 
 890                          done = (num_points > 5*loop_step or num_points > max_num_points) 
 891                          done = done and not \ 
 892                              check_bounds(array([P_y['null_curve_y'].new_sol_segment[xname], 
 893                                                  P_y['null_curve_y'].new_sol_segment[yname]]).T, 
 894                                           xinterval, yinterval) 
 895                      else: 
 896                          # did not start in sub-domain and still not yet found 
 897                          # it during continuation 
 898                          y_null_part = crop_2D(array([P_y['null_curve_y'].sol[xname], 
 899                                                       P_y['null_curve_y'].sol[yname]]).T, 
 900                                                xinterval, yinterval) 
 901                          in_subom = len(y_null_part)>0 
 902                          done = num_points > 15*loop_step 
 903   
 904              # overwrite y_null from fsolve, pre-PyCont 
 905              # can get repetition of some points 
 906              if strict_domains: 
 907                  y_null = crop_2D(array([P_y['null_curve_y'].sol[xname], 
 908                                      P_y['null_curve_y'].sol[yname]]).T, 
 909                               xinterval, yinterval) 
 910              else: 
 911                  y_null = array([P_y['null_curve_y'].sol[xname], 
 912                                  P_y['null_curve_y'].sol[yname]]).T 
 913              try: 
 914                  x_vals = y_null[:,0] 
 915                  y_vals = y_null[:,1] 
 916              except IndexError: 
 917                  raise PyDSTool_ExistError('null_curve_y failed: points were out of domain') 
 918              y_null = uniquePoints(y_null) 
 919              # add fixed points to nullcline, assuming sufficient accuracy 
 920              fp_ixs = [findClosestPointIndex(pt, y_null) for pt in add_fp_pts] 
 921              for n, ix in enumerate(fp_ixs): 
 922                  # +n offsets fact that n entries were already added 
 923                  y_null = np.insert(y_null, ix+n, add_fp_pts[n], axis=0) 
 924   
 925          # X 
 926          if xname in do_vars: 
 927              # set up a prioritized list of starting points 
 928              x_init_priority_list = [] 
 929              y_init_priority_list = [] 
 930              if fps is not None and len(fps) > 0: 
 931                  x_init = fps[0][xname_orig] + 1e-4*(x_dom[1]-x_dom[0]) 
 932                  y_init = fps[0][yname_orig] + 1e-4*(y_dom[1]-y_dom[0]) 
 933                  x_init_priority_list.append(x_init) 
 934                  y_init_priority_list.append(y_init) 
 935              if len(x_null_fsolve) > 0: 
 936                  # get an initial point from user-specified sub-domain 
 937                  index = int(len(x_null_fsolve)/2.) 
 938                  x_init, y_init = x_null_fsolve[index] 
 939                  x_init_priority_list.append(x_init) 
 940                  y_init_priority_list.append(y_init) 
 941              x_init_priority_list.extend( seed_pts_x_for_xnull ) 
 942              y_init_priority_list.extend( seed_pts_y_for_xnull ) 
 943              if len(x_null_inh) > 0: 
 944                  old_indices = [] 
 945                  for ratio in (0.25, 0.5, 0.75): 
 946                      index = int(len(x_null_inh)*ratio) 
 947                      if index in old_indices: 
 948                          continue 
 949                      else: 
 950                          old_indices.append(index) 
 951                      x_init, y_init = x_null_inh[index] 
 952                      x_init_priority_list.append(x_init) 
 953                      y_init_priority_list.append(y_init) 
 954              # lowest priority: domain midpoint 
 955              x_init_priority_list.append( (x_dom[0]+x_dom[1])/2. ) 
 956              y_init_priority_list.append( (y_dom[0]+y_dom[1])/2. ) 
 957   
 958              # initialize starting point 
 959              x_init = x_init_priority_list[0] 
 960              y_init = y_init_priority_list[0] 
 961   
 962              max_tries = min(len(x_init_priority_list), 4) 
 963   
 964              if pycont_cache[0] is None: 
 965                  sysargs_x = args(name='nulls_x', pars={yname: y_init}, 
 966                             ics={xname: x_init}, tdata=[t,t+1], 
 967                             xdomain={xname: gen.xdomain[xname]}, pdomain={yname: gen.xdomain[yname]}) 
 968                  if 'inputs' in gen.funcspec._initargs: # and isinstance(gen.funcspec._initargs['inputs'], dict): 
 969                      if len(gen.funcspec._initargs['inputs']) > 0: 
 970                          sysargs_x['inputs'] = gen.inputs.copy() 
 971                  sysargs_x.pars.update(gen.pars) 
 972                  sysargs_x.pars.update(filteredDict(vardict, [xname, yname], neg=True)) 
 973                  varspecs = {xname: gen.funcspec._initargs['varspecs'][xname]} 
 974                  if 'fnspecs' in gen.funcspec._initargs: 
 975                      old_fnspecs = gen.funcspec._initargs['fnspecs'] 
 976                      fnspecs, new_varspecs = resolveClashingAuxFnPars(old_fnspecs, varspecs, 
 977                                                                       sysargs_x.pars.keys()) 
 978                      # TEMP 
 979                      # process any Jacobian functions to remove un-needed terms 
 980                      if 'Jacobian' in fnspecs: 
 981                          del fnspecs['Jacobian'] 
 982  ##                        fnspecs['Jacobian'] = makePartialJac(fnspecs['Jacobian'], [xname]) 
 983  ##                    if 'Jacobian_pars' in fnspecs: 
 984  ##                        fnspecs['Jacobian_pars'] = makePartialJac(fnspecs['Jacobian_pars'], [xname]) 
 985  ##                    elif 'Jacobian' in fnspecs: 
 986  ##                        old_fargs, J_par_spec = makePartialJac(old_fnspecs['Jacobian'], [xname], [yname]) 
 987  ##                        fnspecs['Jacobian_pars'] = (fnspecs['Jacobian'][0], J_par_spec) 
 988                      sysargs_x.update({'fnspecs': fnspecs}) 
 989                  else: 
 990                      new_varspecs = varspecs 
 991                  sysargs_x.varspecs = new_varspecs 
 992                  sysargs_x.pars['time'] = t 
 993                  sys_x = Vode_ODEsystem(sysargs_x) 
 994                  P_x = ContClass(sys_x) 
 995                  pycont_cache[0] = P_x 
 996              else: 
 997                  P_x = pycont_cache[0] 
 998                  new_pars = gen.pars.copy() 
 999                  new_pars.update(filteredDict(vardict, [xname, yname], neg=True)) 
1000                  new_pars['time'] = t 
1001                  P_x.model.set(pars=new_pars) 
1002              PCargs = args(name='null_curve_x', type='EP-C', force=True) 
1003              PCargs.freepars = [yname] 
1004              PCargs.MinStepSize = 1e-8 
1005              PCargs.VarTol = PCargs.FuncTol = eps*10 
1006              PCargs.TestTol = eps 
1007              if max_step is None: 
1008                  PCargs.MaxStepSize = 5e-1 
1009                  PCargs.StepSize = 1e-6 
1010              else: 
1011                  PCargs.MaxStepSize = max_step[yname] 
1012                  PCargs.StepSize = max_step[yname]*0.5 
1013              PCargs.MaxNumPoints = loop_step 
1014              PCargs.MaxCorrIters = 8 
1015              #PCargs.verbosity = 100 
1016              P_x.newCurve(PCargs) 
1017              done = False 
1018              num_points = 0 
1019              in_subdom = x_init in xinterval and y_init in yinterval 
1020              tries = 0 
1021              while not done: 
1022                  try: 
1023                      P_x['null_curve_x'].forward() 
1024                  except PyDSTool_ExistError: 
1025                      tries += 1 
1026                      if tries == max_tries: 
1027                          print 'null_curve_x failed in forward direction' 
1028                          raise 
1029                      else: 
1030                          # try a different starting point 
1031                          x_init = x_init_priority_list[tries] 
1032                          y_init = y_init_priority_list[tries] 
1033                          sys_x.set(pars={yname: y_init}, ics={xname: x_init}) 
1034                  else: 
1035                      num_points += loop_step 
1036                      # stop if have tried going forwards more than 5 times and solution is 
1037                      # still not within domain, or if num_points exceeds max_num_points 
1038                      done = (num_points > 5*loop_step and not \ 
1039                              check_bounds(array([P_x['null_curve_x'].new_sol_segment[xname], 
1040                                                    P_x['null_curve_x'].new_sol_segment[yname]]).T, 
1041                                              xinterval, yinterval)) \ 
1042                           or num_points > max_num_points 
1043              done = False 
1044              num_points = 0 
1045              in_subdom = x_init in xinterval and y_init in yinterval 
1046              tries = 0 
1047              while not done: 
1048                  try: 
1049                      P_x['null_curve_x'].backward() 
1050                  except PyDSTool_ExistError: 
1051                      tries += 1 
1052                      if tries == max_tries: 
1053                          print 'null_curve_x failed in backward direction' 
1054                          raise 
1055                      else: 
1056                          # try a different starting point 
1057                          x_init = x_init_priority_list[tries] 
1058                          y_init = y_init_priority_list[tries] 
1059                          sys_x.set(pars={yname: y_init}, ics={xname: x_init}) 
1060                  else: 
1061                      num_points += loop_step 
1062                      # stop if have tried going backwards more than 5 times and solution is 
1063                      # still not within domain, or if num_points exceeds max_num_points 
1064                      done = (num_points > 5*loop_step and not \ 
1065                              check_bounds(array([P_x['null_curve_x'].new_sol_segment[xname], 
1066                                                    P_x['null_curve_x'].new_sol_segment[yname]]).T, 
1067                                              xinterval, yinterval)) \ 
1068                           or num_points > max_num_points 
1069   
1070              # overwrite x_null from fsolve, pre-PyCont 
1071              # can get repetition of some points 
1072              if strict_domains: 
1073                  x_null = crop_2D(array([P_x['null_curve_x'].sol[xname], 
1074                                          P_x['null_curve_x'].sol[yname]]).T, 
1075                               xinterval, yinterval) 
1076              else: 
1077                  x_null = array([P_x['null_curve_x'].sol[xname], 
1078                                  P_x['null_curve_x'].sol[yname]]).T 
1079              try: 
1080                  x_vals = x_null[:,0] 
1081              except IndexError: 
1082                  raise PyDSTool_ExistError('null_curve_x failed: points were out of domain') 
1083              x_null = uniquePoints(x_null) 
1084              # add fixed points to nullcline, assuming sufficient accuracy 
1085              fp_ixs = [findClosestPointIndex(pt, x_null) for pt in add_fp_pts] 
1086              for n, ix in enumerate(fp_ixs): 
1087                  # +n offsets fact that n entries were already added 
1088                  x_null = np.insert(x_null, ix+n, add_fp_pts[n], axis=0) 
1089   
1090      else: 
1091          x_null = x_null_fsolve 
1092          y_null = y_null_fsolve 
1093      if do_cache: 
1094          return (gen._FScompatibleNamesInv(x_null), gen._FScompatibleNamesInv(y_null), pycont_cache) 
1095      else: 
1096          return (gen._FScompatibleNamesInv(x_null), gen._FScompatibleNamesInv(y_null)) 
1097   
1098   
1099   


1100 -def find_fixedpoints(gen, subdomain=None, n=5, maxsearch=1e3, eps=1e-8, 
1101                       t=0, jac=None):


1102      """Find fixed points of a system in a given sub-domain (dictionary), 
1103      on the assumption that they are isolated points. subdomain may contain 
1104      pairs (min, max) or singleton values that fix those state variables 
1105      and restrict the fixed point search to the remaining sub-system on the 
1106      given ranges. (default to domains given in generator). 
1107   
1108      Returns list of dictionaries mapping the variable names to the values. 
1109   
1110      Set t value for non-autonomous systems (default 0). 
1111      """ 
1112      # get state variable domains if subdomain dictionary not given 
1113      if subdomain is None: 
1114          subdomain = filteredDict(gen.xdomain, gen.funcspec.vars) 
1115      else: 
1116          subdomain = gen._FScompatibleNames(subdomain) 
1117          assert remain(subdomain.keys(),gen.funcspec.vars) == [] and \ 
1118                 remain(gen.funcspec.vars,subdomain.keys()) == [] 
1119      # only vary over domains that are given as ranges: fixed values 
1120      # are not counted 
1121      D = 0 
1122      xdict = {}.fromkeys(gen.funcspec.vars) 
1123      fixed_vars = {} 
1124      for xname, dom in subdomain.iteritems(): 
1125          if isinstance(dom, (tuple,list)): 
1126              if not (isfinite(dom[0]) and isfinite(dom[1])): 
1127                  raise RuntimeError("Must specify a finite range for %s"%xname) 
1128              D += 1 
1129          else: 
1130              xdict[xname] = dom 
1131              # put fixed vars back into return value 
1132              fixed_vars[xname] = dom 
1133      var_ix_map = invertMap(gen.funcspec.vars) 
1134      # pick n uniformly distributed starting points in domains, 
1135      # ensuring n isn't so large as to require more than maxsearch 
1136      # number of searches 
1137      while True: 
1138          if n**D > maxsearch: 
1139              n = n-1 
1140          else: 
1141              break 
1142          if n < 3: 
1143              raise PyDSTool_ValueError("maxsearch too small") 
1144      x0_coords = np.zeros((D,n),'f') 
1145      x0_ixs = [] 
1146      x0_names = [] 
1147      ix = 0 
1148      xintervals = [] 
1149      # sort names by key to ensure same ordering as generator variables 
1150      for xname, xdom in sortedDictItems(subdomain, byvalue=False): 
1151          if isinstance(xdom, (tuple, list)): 
1152              xintervals.append( Interval('xdom', float, subdomain[xname]) ) 
1153              x0_ixs.append(var_ix_map[xname]) 
1154              x0_names.append(xname) 
1155              x0_coords[ix,:] = linspace(xdom[0], xdom[1], n) 
1156              ix += 1 
1157      # NOTE: def Rhs(self, t, xdict, pdict) and Jacobian signature 
1158      # has same form, so need to use a wrapper function to convert order 
1159      # of arguments to suit solver. 
1160      # 
1161      Rhs_wrap = make_RHS_wrap(gen, xdict, x0_names) 
1162      if gen.haveJacobian(): 
1163          fprime = make_Jac_wrap(gen, xdict, x0_names) 
1164      elif jac is not None: 
1165          def Jac_wrap(x, t, pdict): 
1166              xdict.update(dict(zip(x0_names, x))) 
1167              argdict = filteredDict(xdict, jac._args) 
1168              argdict['t'] = t 
1169              try: 
1170                  return array(jac(**argdict)) 
1171              except (OverflowError, ValueError): 
1172                  # penalty 
1173                  return array([[1e4]*D]*D)

1174          fprime = Jac_wrap 
1175      else: 
1176          fprime = None 
1177   
1178      # solve xdot on each starting point 
1179      fps = [] 
1180      fp_listdict = [] 
1181      d_posns = base_n_counter(n,D) 
1182      xtol = eps/10. 
1183      for dummy_ix in xrange(n**D): 
1184          x0 = array([x0_coords[i][d_posns[i]] for i in xrange(D)]) 
1185          res = fsolve(Rhs_wrap,x0,(t,gen.pars),xtol=xtol, 
1186                            fprime=fprime,full_output=True) 
1187          xinf_val = res[0] 
1188          if D == 1: 
1189              xinf_val = array([xinf_val]) 
1190          # treat f.p.s within epsilon (2-norm) of each other as identical 
1191          if alltrue(isfinite(xinf_val)): 
1192              if len(fps) == 0 or not sometrue([norm(fp-xinf_val)<eps for fp in fps]): 
1193                  ok = res[2]==1 
1194                  # check that bounds were met 
1195                  if ok: 
1196                      for ix, xname in enumerate(x0_names): 
1197                          ok = ok and \ 
1198                            xintervals[ix].contains(xinf_val[ix]) is not notcontained 
1199                  if ok: 
1200                      fps.append(xinf_val) 
1201                      fp_pt = dict(zip(x0_names, xinf_val)) 
1202                      fp_pt.update(fixed_vars) 
1203                      fp_listdict.append(gen._FScompatibleNamesInv(fp_pt)) 
1204          d_posns.inc() 
1205      return tuple(fp_listdict) 
1206   
1207   
1208  # convenient aliases 
1209  find_steadystates = find_equilibria = find_fixedpoints 
1210   
1211   


1212 -class Point2D(Point):


1213      """Convenience sub-class of PyDSTool.Point for 2D Euclidean points. 
1214      If initialized using a dictionary, xname and yname optional arguments 
1215      must specify which coordinate is associated with 'x' and 'y' axes. 
1216   
1217      Main advantage is lower overhead of initialization and the more convenient 
1218      notation point.x and point.y for the two coordinates, that may 
1219      nonetheless be given optional actual names (xname and yname). Note that 
1220      Python's "dot-based" attribute lookup is only valid for 'x' and 'y' fields. 
1221   
1222      Optional norm order and labels argument still permitted, as for original 
1223      Point. For 2D Pointsets, use original PyDSTool.Pointset class. 
1224      """ 


1225 -    def __init__(self, x, y=None, xname='x', yname='y', norm=2, labels=None):


1226          """If y is None, expects 2D array or dictionary in x""" 
1227          if y is None: 
1228              try: 
1229                  # array x? 
1230                  self.x = float(x[0]) 
1231                  self.y = float(x[1]) 
1232              except (TypeError, KeyError): 
1233                  # dictionary x? 
1234                  self.x = float(x[xname]) 
1235                  self.y = float(x[yname]) 
1236          else: 
1237              self.x = float(x) 
1238              self.y = float(y) 
1239          self.xname = xname 
1240          self.yname = yname 
1241          self._parameterized = False 
1242          if labels is None: 
1243              self.labels = {} 
1244          else: 
1245              self.addlabel(labels) 
1246          self._normord = norm 
1247          self.coordtype = float 
1248          self.dimension = 2 
1249          self.coordarray = None  # Not implemented this way

1250   


1251 -    def mapNames(self, themap):


1252          self.xname = themap(self.xname) 
1253          self.yname = themap(self.yname) 
1254          self.labels = mapNames(themap, self.labels)

1255   


1256 -    def __contains__(self, coord):


1257          return coord in (self.xname, self.yname)

1258   


1259 -    def todict(self, aslist=False):


1260          """Convert Point2D to a dictionary of array values (or of list with aslist=True).""" 
1261          if aslist: 
1262              return {self.xname: [self.x], 
1263                      self.yname: [self.y]} 
1264          else: 
1265              return {self.xname: self.x, 
1266                      self.yname: self.y}

1267   


1268 -    def toarray(self):


1269          return np.array( (self.x, self.y) )

1270   


1271 -    def toPoint(self):


1272          """Coerce to regular PyDSTool.Point object""" 
1273          return Point(coorddict={xname: self.x, yname: self.y}, 
1274                       coordtype=float, 
1275                       norm=self._normord, 
1276                       labels=self.labels)

1277   


1278 -    def get(self, coord, d=None):


1279          if coord == self.xname: 
1280              return self.x 
1281          elif coord == self.yname: 
1282              return self.y 
1283          else: 
1284              return d

1285   


1286 -    def __len__(self):


1287          return 2

1288   


1289 -    def __abs__(self):


1290          return np.linalg.norm( (self.x, self.y), self._normord)

1291   


1292 -    def update(self, d):


1293          self.x = d[self.xname] 
1294          self.y = d[self.yname]

1295   


1296 -    def items(self):


1297          return ((self.xname, self.x), (self.yname, self.y))

1298   


1299 -    def iteritems(self):


1300          return iter(((self.xname, self.x), (self.yname, self.y)))

1301   


1302 -    def values(self):


1303          return [self.x, self.y]

1304   


1305 -    def itervalues(self):


1306          return iter([self.x, self.y])

1307   


1308 -    def keys(self):


1309          return [self.xname, self.yname]

1310   


1311 -    def iterkeys(self):


1312          return iter([self.xname, self.yname])

1313   


1314 -    def has_key(self, k):


1315          return k in (self.xname, self.yname)

1316   


1317 -    def __getitem__(self, ix):


1318          if ix == 0: 
1319              return self.x 
1320          elif ix == 1: 
1321              return self.y 
1322          elif isinstance(ix, slice): 
1323              return (self.x, self.y) 
1324          else: 
1325              raise StopIteration #IndexError("Index out of range")

1326   
1327      __call__ = __getitem__ 
1328   


1329 -    def __setitem__(self, ix, val):


1330          if ix == 0: 
1331              self.x = val 
1332          elif ix == 1: 
1333              self.y = val 
1334          else: 
1335              raise IndexError("Index out of range")

1336   


1337 -    def __delitem__(self, k):


1338          raise NotImplementedError

1339   


1340 -    def __str__(self):


1341          return "Point2D( %f, %f )" % (self.x, self.y)

1342   


1343 -    def __repr__(self):


1344          if self.labels == {}: 
1345              labstr = 'no labels' 
1346          else: 
1347              labstr = 'labels' 
1348          return "Point2D( %f, %f, %s, %s, %i, %s )" % (self.x, self.y, 
1349                              self.xname, self.yname, self._normord, labstr)

1350   


1351 -    def info(self, verboselevel=1):


1352          if verboselevel == 1: 
1353              print self.__str__() 
1354          elif verboselvel > 1: 
1355              print self.__repr__()

1356   


1357 -    def __copy__(self):


1358          return Point2D(self.x, self.y, self.xname, self.yname, self._normord, 
1359                         self.labels)

1360   


1361 -    def __add__(self, other):


1362          try: 
1363              return Point2D( self.x + other[0], self.y + other[1], 
1364                              self.xname, self.yname, self._normord, self.labels) 
1365          except TypeError: 
1366              return Point2D( self.x + other, self.y + other, 
1367                              self.xname, self.yname, self._normord, self.labels )

1368   


1369 -    def __sub__(self, other):


1370          try: 
1371              return Point2D( self.x - other[0], self.y - other[1], 
1372                              self.xname, self.yname, self._normord, self.labels ) 
1373          except TypeError: 
1374              return Point2D( self.x - other, self.y - other, 
1375                              self.xname, self.yname, self._normord, self.labels )

1376   
1377      __radd__ = __add__ 
1378   


1379 -    def __rsub__(self, other):


1380          try: 
1381              return Point2D( other[0] - self.x, other[1] - self.y, 
1382                              self.xname, self.yname, self._normord, self.labels ) 
1383          except TypeError: 
1384              return Point2D( other - self.x, other - self.y, 
1385                              self.xname, self.yname, self._normord, self.labels )

1386   


1387 -    def __mul__(self, c):


1388          # scalar 
1389          return Point2D( self.x * c, self.y * c, 
1390                              self.xname, self.yname, self._normord, self.labels)

1391   


1392 -    def __div__(self, c):


1393          # scalar 
1394          return Point2D( self.x / float(c), self.y / float(c), 
1395                              self.xname, self.yname, self._normord, self.labels )

1396   


1397 -    def __rmul__(self, c):


1398          # scalar 
1399          return Point2D( self.x * c, self.y * c, 
1400                              self.xname, self.yname, self._normord, self.labels)

1401   


1402 -    def __rdiv__(self, c):


1403          # scalar 
1404          return Point2D( float(c) / self.x, float(c) / self.y, 
1405                              self.xname, self.yname, self._normord, self.labels )

1406   
1407      __truediv__ = __div__ 
1408      __rtruediv__ = __rdiv__ 
1409   


1410 -    def __neg__(self):


1411          return Point2D( -self.x, -self.y, 
1412                              self.xname, self.yname, self._normord, self.labels )

1413   


1414 -    def __pow__(self, other):


1415          return Point2D( self.x ** other, self.y ** other, 
1416                              self.xname, self.yname, self._normord, self.labels )

1417   


1418 -    def __eq__(self, other):


1419          return self.x == other[0] and self.y == other[1]

1420   


1421 -    def __lt__(self, other):


1422          try: 
1423              return np.linalg.norm( (self.x, self.y), self._normord ) < \ 
1424                     np.linalg.norm( (other.x, other.y), self._normord ) 
1425          except AttributeError: 
1426              # scalar or array 
1427              return np.linalg.norm( (self.x, self.y), self._normord ) < \ 
1428                     np.linalg.norm( other, self._normord )

1429   


1430 -    def __gt__(self, other):


1431          try: 
1432              return np.linalg.norm( (self.x, self.y), self._normord ) > \ 
1433                     np.linalg.norm( (other.x, other.y), self._normord ) 
1434          except AttributeError: 
1435              # scalar or array 
1436              return np.linalg.norm( (self.x, self.y), self._normord ) > \ 
1437                     np.linalg.norm( other, self._normord )

1438   


1439 -    def __le__(self, other):


1440          try: 
1441              return np.linalg.norm( (self.x, self.y), self._normord ) <= \ 
1442                     np.linalg.norm( (other.x, other.y), self._normord ) 
1443          except AttributeError: 
1444              # scalar or array 
1445              return np.linalg.norm( (self.x, self.y), self._normord ) <= \ 
1446                     np.linalg.norm( other, self._normord )

1447   


1448 -    def __ge__(self, other):


1449          try: 
1450              return np.linalg.norm( (self.x, self.y), self._normord ) >= \ 
1451                     np.linalg.norm( (other.x, other.y), self._normord ) 
1452          except AttributeError: 
1453              # scalar or array 
1454              return np.linalg.norm( (self.x, self.y), self._normord ) >= \ 
1455                     np.linalg.norm( other, self._normord )

1456   
1457   


1458 -class nullcline(object):


1459      """Nullcline representation class in 2D (x, y) plane parameterizable 
1460      by x variable only. A third-order univariate spline will be fitted to 
1461      the given sample point array using all as knots, and available 
1462      through the 'spline' attribute. 
1463   
1464      Input: 
1465        Names are given to the x and y axis directions at initialization. 
1466        Third argument nullc_array is the N-by-2 array of N two-dimensional 
1467          data points. 
1468        The final, optional argument x_relative_scale indicates the relative 
1469          scale of the x values' domain compared to the y values. This is 
1470          used to provide a form of subjective normalization for measuring 
1471          curvature and other geometric properties when plotting y vs. x 
1472          on these scales. E.g if x values range over -50 to +50, while 
1473          y ranges over 0 to 1, x_relative_scale=100 is appropriate. 
1474   
1475      N.B.: Only works for nullclines that can be written y = f(x). Future 
1476      versions may support non-functions. 
1477   
1478      Some functions acting on nullclines currently also rely on monotonicity 
1479      of y=f(x), and will verify this using the is_monotonic method before 
1480      proceeding. Future versions will attempt to resolve that. 
1481      """ 


1482 -    def __init__(self, xname, yname, nullc_array, x_relative_scale=1):


1483          self.xname = xname 
1484          self.yname = yname 
1485          self.array = nullc_array 
1486          # ensure monotonicity 
1487          if not isincreasing(nullc_array[:,0]): 
1488              raise AssertionError("x axis '%s' values must be monotonically increasing" % xname) 
1489          self.spline = InterpolatedUnivariateSpline(nullc_array[:,0], 
1490                                                     nullc_array[:,1]) 
1491          # store these precomputed values in advance for efficiency 
1492          self.x_relative_scale_fac = x_relative_scale 
1493          self.x_relative_scale_fac_2 = self.x_relative_scale_fac**2 
1494          self.x_relative_scale_fac_3 = self.x_relative_scale_fac**3

1495   


1496 -    def __call__(self, x):


1497          """Returns a scalar if x is scalar.""" 
1498          if isinstance(x, _num_types): 
1499              return self.spline(x)[0] 
1500          else: 
1501              return self.spline(x)

1502   


1503 -    def createParameterization(self):


1504          """Creates a (non-arc-length) parameterization""" 
1505          raise NotImplementedError

1506   


1507 -    def toPointset(self, with_param=False):


1508          if with_param: 
1509              self.createParameterization() 
1510              #return arrayToPointset(x_vals, y_vals, self.xname, self.yname, params, 's') 
1511          else: 
1512              return Pointset({self.xname: self.array[:,0], 
1513                               self.yname: self.array[:,1]})

1514   


1515 -    def sample_x_domain(self, refine=0):


1516          return _sample_array_interior(self.array[:,0], 0.01, 
1517                                                       refine=refine)

1518   


1519 -    def tgt_vec(self, x, rescale=False):


1520          """Return tangent vector to an interior point of nullcline, 
1521          normalized to length 1. Set rescale option (default False) 
1522          to return a vector rescaled according to relative x vs. y scales 
1523          (thus it's no longer a tangent vector in that case).""" 
1524          try: 
1525              der = self.spline(x,1)  # first deriv through option to __call__ 
1526          except AssertionError: 
1527              raise ValueError("Derivative not available for endpoints or outside of spline domain") 
1528          else: 
1529              if rescale: 
1530                  tgt = np.array((1, self.x_relative_scale_fac_2*der)) 
1531              else: 
1532                  tgt = np.array((1, der)) 
1533          return tgt/np.linalg.norm(tgt)

1534   


1535 -    def curvature(self, xdata):


1536          """Signed curvature at x is returned (scalar) for scalar x, 
1537          otherwise array of such scalars for 1D array x. 
1538   
1539          Positive values mean concave "up" in the plane.""" 
1540          try: 
1541              ders = np.array([self.spline.derivatives(x) for x in xdata]) 
1542          except AssertionError: 
1543              raise ValueError("Derivative not available for endpoints or " 
1544                               "outside of spline domain") 
1545          except TypeError: 
1546              # singleton 
1547              try: 
1548                  ders = self.spline.derivatives(xdata) 
1549              except AssertionError: 
1550                  raise ValueError("Derivative not available for endpoints " 
1551                                   "or outside of spline domain") 
1552              return self.x_relative_scale_fac_2*ders[2] / \ 
1553                     pow(1+self.x_relative_scale_fac_2*ders[1]*ders[1],1.5) 
1554          else: 
1555              return self.x_relative_scale_fac_2*ders[:,2] / \ 
1556                     np.power(1+self.x_relative_scale_fac_2*ders[:,1]*ders[:,1],1.5)

1557   


1558 -    def grad_curvature(self, xdata):


1559          """Derivative of the curvature at x is returned (scalar) for scalar x, 
1560          otherwise array of such scalars for 1D array x.""" 
1561          try: 
1562              ders = np.array([self.spline.derivatives(x) for x in xdata]) 
1563          except AssertionError: 
1564              raise ValueError("Derivative not available for endpoints or " 
1565                               "outside of spline domain") 
1566          except TypeError: 
1567              # singleton 
1568              try: 
1569                  ders = self.spline.derivatives(xdata) 
1570              except AssertionError: 
1571                  raise ValueError("Derivative not available for endpoints " 
1572                                   "or outside of spline domain") 
1573              denom = pow(1+self.x_relative_scale_fac_2*ders[1]*ders[1],2.5) 
1574              return (ders[3]*self.x_relative_scale_fac_2*(1+self.x_relative_scale_fac_2*ders[1]*ders[1]) - \ 
1575                     3*(self.x_relative_scale_fac_2*self.x_relative_scale_fac_2*ders[2]*ders[2]*ders[1])) / denom 
1576          else: 
1577              d1_sq = ders[:,1]*ders[:,1] 
1578              denom = np.power(1+self.x_relative_scale_fac_2*d1_sq,2.5) 
1579              return (ders[:,3]*self.x_relative_scale_fac_2*(1+self.x_relative_scale_fac_2*d1_sq) - \ 
1580                     3*(self.x_relative_scale_fac_2*self.x_relative_scale_fac_2*ders[:,2]*ders[:,2]*ders[:,1])) / denom

1581   


1582 -    def curvature_at_sample_points(self, refine=0):


1583          """Returns array of signed scalars for each 
1584          x data point (knot) of the nullcline spline (computed just 
1585          inside endpoints by 1% of distance to next innermost sample 
1586          point). 
1587   
1588          Positive values mean concave "up" in the plane. 
1589          Assumes nullcline is monotonic.""" 
1590          return self.curvature(_sample_array_interior(self.array[:,0], 0.01, 
1591                                                       refine=refine))

1592   


1593 -    def grad_curvature_at_sample_points(self, refine=0):


1594          """Returns array of signed scalars for each 
1595          x data point (knot) of the nullcline spline (computed just 
1596          inside endpoints by 1% of distance to next innermost sample 
1597          point). 
1598   
1599          Assumes nullcline is monotonic.""" 
1600          return self.grad_curvature(_sample_array_interior(self.array[:,0], 
1601                                                            0.01, refine=refine))

1602   


1603 -    def concavity(self, xdata):


1604          """Concavity scalar +/- 1 or 0 at x is returned for scalar x, 
1605          otherwise array of such scalars for 1D array x. 
1606   
1607          Positive values mean concave "up" in the plane.""" 
1608          return np.sign(self.curvature(xdata))

1609   


1610 -    def concavity_at_sample_points(self, refine=0):


1611          """Returns array of +/- 1 or 0 scalars for each 
1612          x data point (knot) of the nullcline spline (computed just 
1613          inside endpoints by 1% of distance to next innermost sample 
1614          point). 
1615   
1616          Positive values mean concave "up" in the plane. 
1617          Assumes nullcline is monotonic.""" 
1618          return self.concavity(_sample_array_interior(self.array[:,0], 0.01, 
1619                                                       refine=refine))

1620   


1621 -    def crop(self, xdom, ydom, resample_frac=0.1):


1622          """Returns a new Nullcline object cropped to the (x, y) domain 
1623          given by pairs xdom and ydom. 
1624   
1625          If resample_frac > 0 (default 0.1), points at the domain 
1626          ends and additional sample points will be included in the new nullcline 
1627          object regardless of whether they were in the original sample point set 
1628          for the cropped nullcline. New points will be selected at the rate 
1629          indicated, measured as a fraction of the domain width. Points that are 
1630          as close as 1/10th of this rate to existing sample points will be 
1631          excluded, to avoid ill-conditioning the spline shape in the resampling. 
1632   
1633          Given the assumption that nullclines are montonic functions of x, 
1634          this guarantees a unique, contiguous piece of the nullcline is returned. 
1635          """ 
1636          xinterval = Interval('xdom', float, xdom) 
1637          yinterval = Interval('ydom', float, ydom) 
1638          sample_vals = list(crop_2D(self.array, xinterval, yinterval)) 
1639          if len(sample_vals) == 0: 
1640              # force resampling if not already used or if too sparse 
1641              if resample_frac == 0 or resample_frac > 0.2: 
1642                  resample_frac = 0.1 
1643              #raise ValueError("No nullcline sample points in given region") 
1644          if resample_frac > 0: 
1645              width = xdom[1] - xdom[0] 
1646              new_xs = xinterval.sample(dt=width*resample_frac) 
1647              tol = width*resample_frac*0.1 
1648              for x in new_xs: 
1649                  # exclude any that are within 5% of domain extent of 
1650                  # existing sample points and within original point extents 
1651                  if np.all(abs(x - self.array[:,0])>tol) and \ 
1652                     x >= self.array[0,0] and x <= self.array[-1,0]: 
1653                      y = self(x) 
1654                      if yinterval.contains(y) is not notcontained: 
1655                          sample_vals.append((x,y)) 
1656              sample_vals = np.array(sample_vals) 
1657              ixs = argsort(sample_vals[:,0])  # sort by x 
1658              sample_vals = sample_vals[ixs] 
1659          try: 
1660              return nullcline(self.xname, self.yname, np.array(sample_vals), 
1661                               x_relative_scale=self.x_relative_scale_fac) 
1662          except: 
1663              print "Error cropping nullcline at sample points", sample_vals 
1664              print "MAYBE TOO FEW VALUES SAMPLED: number was", len(sample_vals) 
1665              raise

1666   


1667 -    def is_monotonic(self):


1668          return ismonotonic(self.array[:,1])

1669   


1670 -    def __copy__(self):


1671          return nullcline(self.xname, self.yname, self.array)

1672   
1673   


1674 -def get_perp(v):


1675      """Find perpendicular vector in 2D (assumes 2D input)""" 
1676      vperp=v.copy()  # ensures correct return type 
1677      vperp[0] = v[1] 
1678      vperp[1] = -v[0] 
1679      return vperp

1680   


1681 -def get_orthonormal(v):


1682      """Returns orthonormal vector in 2D (assumes 2D input)""" 
1683      vperp=v.copy()  # ensures correct return type 
1684      vperp[0] = v[1] 
1685      vperp[1] = -v[0] 
1686      return vperp/np.linalg.norm(vperp)

1687   


1688 -def get_rotated(x, theta):


1689      res = copy.copy(x) # ensure same type of result as input 
1690      z=(x[0]+x[1]*1j)*(cos(theta)+sin(theta)*1j) 
1691      res[0] = z.real 
1692      res[1] = z.imag 
1693      return res

1694   


1695 -def filter_close_points(pts, eps, normord=2):


1696      """Remove points from iterable pts (e.g. array or Pointset) 
1697      according to whether they are closer than epsilon to each other 
1698      in the given norm. 
1699      """ 
1700      # start with all indices, and remove those that are unwanted 
1701      remaining = range(len(pts)) 
1702      for i, p in enumerate(pts): 
1703          for j in range(i+1, len(pts)): 
1704              if norm(p-pts[j],normord) < eps: 
1705                  try: 
1706                      remaining.remove(j) 
1707                  except ValueError: 
1708                      # already removed 
1709                      pass 
1710      return pts[remaining]

1711   
1712   


1713 -def filter_NaN(pts):


1714      """Filter out any points containing NaNs. 
1715      Expects array input. 
1716      """ 
1717      return array([p for p in pts if all(isfinite(p))])

1718   


1719 -def check_bounds(sol_array, xinterval, yinterval):


1720      # x is assumed to be the dynamic variable 
1721      return alltrue([xinterval.contains(p[0]) is not notcontained and \ 
1722                      yinterval.contains(p[1]) is not notcontained for p in sol_array])

1723   


1724 -def crop_2D(sol_array, xinterval, yinterval):


1725      """Filter out points that are outside the domains given by xdom, ydom. 
1726      Expects array input 
1727      """ 
1728      return array([p for p in sol_array if xinterval.contains(p[0]) is not notcontained \ 
1729                and yinterval.contains(p[1]) is not notcontained])

1730   


1731 -def make_vec_at_A_face_B(vec_at_a, Ay, By):


1732      """Assumes monotonicity of functions on which points A and B lie, 
1733      using their y components as arguments.""" 
1734      up = np.array( (0.0,1.0) ) 
1735      orient = 1   # default value 
1736      if Ay > By: 
1737          # A is above B so vec must orient "down" 
1738          if np.dot( up, vec_at_a ) > 0: 
1739              # vec is up 
1740              orient = -1 
1741      else: 
1742          # B is above A so vec must orient "up" 
1743          if np.dot( up, vec_at_a ) < 0: 
1744              # vec is down 
1745              orient = -1 
1746      return orient * vec_at_a

1747   
1748   


1749 -def angle_to_vertical(v):


1750      """Return an angle between 0 and 2*pi measured clockwise from vertical.""" 
1751      up = np.array((0.,1.)) 
1752      theta = np.arccos(np.dot(up, v)/np.linalg.norm(v)) 
1753      if np.cross(up, v) > 0: 
1754          # angle will be greater than pi 
1755          return 2*np.pi - theta 
1756      else: 
1757          return theta

1758   
1759   
1760  # not currently used, but helpful if lines are not arranged correctly for intersection test 


1761 -def is_counter_clockwise(A,B,C):


1762      return (C.y-A.y)*(B.x-A.x) > (B.y-A.y)*(C.x-A.x)

1763   
1764   


1765 -def determine_if_intersect(A,B,C,D):


1766      ccw = is_counter_clockwise 
1767      return ccw(A,C,D) != ccw(B,C,D) and ccw(A,B,C) != ccw(A,B,D)

1768   
1769   


1770 -def line_intersection(A, B, C, D):


1771      """Assumes line segments AB vs. CD actually cross (must determine this 
1772      separately), where points A - D have fields 'x' and 'y' e.g. a Point2D 
1773      object. 
1774   
1775      Uses algorithm from: 
1776      http://www.bryceboe.com/2006/10/23/line-segment-intersection-algorithm/ 
1777      and 
1778      http://paulbourke.net/geometry/lineline2d/ 
1779   
1780      In particular, this code assumes lines are not parallel or collinear. 
1781      """ 
1782      ua =  ( (D.x-C.x)*(A.y-C.y)-(D.y-C.y)*(A.x-C.x) ) / \ 
1783                 ( (D.y-C.y)*(B.x-A.x) - (D.x-C.x)*(B.y-A.y) ) 
1784      return Point2D( A.x+(ua*(B.x-A.x)), A.y+(ua*(B.y-A.y)) )

1785   
1786   


1787 -class fixedpoint_nD(object):


1788      _classifcations = ('spiral', 'node', 'saddle') 
1789      _stability = ('s','c','u') 
1790   


1791 -    def __init__(self, gen, pt, coords=None, jac=None, description='', 
1792                   normord=2, eps=1e-12):


1793          """pt must have same dimension as generator, but if a sub-system 
1794          is being analyzed then specify the sub-system's variables using 
1795          the coords argument. 
1796          """ 
1797          if not isinstance(pt, Point): 
1798              raise PyDSTool_TypeError("Fixed point must be a Point type") 
1799          if coords is None: 
1800              self.dimension = gen.dimension 
1801              self.fp_coords = gen.funcspec.vars 
1802          else: 
1803              self.dimension = len(coords) 
1804              self.fp_coords = coords 
1805          self.eps = eps 
1806          self.gen = gen 
1807          # redefine jac if necessary and allocate to self attribute 
1808          self.jac = jac = self._ensure_jac(jac) 
1809          jac_test_arg = filteredDict(pt, self.fp_coords) 
1810          jac_test_arg['t'] = 0 
1811          if hasattr(jac, '_namemap'): 
1812              # jac made with expr2fun 
1813              self.D = asarray(jac.alt_call(jac_test_arg)) 
1814          else: 
1815              try: 
1816                  self.D = asarray(jac(**jac_test_arg)) 
1817              except: 
1818                  # placeholder 
1819                  raise 
1820          assert self.D.shape == (self.dimension, self.dimension) 
1821          assert normord > 0 
1822          self.normord = normord 
1823          assert pt._normord == normord, "Mismatching norm order for point" 
1824          self._get_eigen() 
1825          evals = self.evals 
1826          evecs = self.evecs 
1827          if self.dimension < gen.dimension: 
1828              subs_str = "sub-" 
1829          else: 
1830              subs_str = "full " 
1831          if self.dimension != len(evals): 
1832              raise ValueError("Dimension of %ssystem must equal number of eigenvalues" % subs_str) 
1833          if self.dimension != len(evecs): 
1834              raise ValueError("Dimension of %ssystem must equal number of eigenvectors" % subs_str) 
1835          if gen.dimension != len(pt): 
1836              raise ValueError("Dimension of full system must equal dimension of fixed point") 
1837          self.point = pt 
1838          # assume autonomous system 
1839          var_ixs = [] 
1840          for v in self.fp_coords: 
1841              var_ixs.append(gen.query('vars').index(v)) 
1842          fp_evaluated = array(gen.Rhs(0, gen._FScompatibleNames(pt), gen.pars))[var_ixs] 
1843          if sometrue([abs(fp_i) > eps for fp_i in fp_evaluated]): 
1844              print "Tolerance =", eps 
1845              print "vector field is", fp_evaluated 
1846              raise PyDSTool_ValueError("Given point is not a fixed point of the system at given tolerance") 
1847          self.coordnames = pt.coordnames 
1848          self._classify() 
1849          self.description = description

1850   


1851 -    def _get_eigen(self):


1852          evals, evecs_array = np.linalg.eig(self.D) 
1853          evecs_pt = [] 
1854          for i, v in enumerate(self.fp_coords): 
1855              d = {} 
1856              for j, w in enumerate(self.fp_coords): 
1857                  # funky indexing is to recover the float value 
1858                  # from a 0-dimensional array (e.g. array(0.5)) 
1859                  # that has no length 
1860                  d[w] = real(evecs_array.T[i][j])[()] 
1861              evecs_pt.append(Point(d)) 
1862          self.evals = evals 
1863          evecs_normed = [] 
1864          for ev in evecs_pt: 
1865              assert ev._normord == self.normord, "Mismatching norm order for eigenvector" 
1866              n = norm(ev, self.normord) 
1867              if n != 1: 
1868                  ev = ev/n 
1869              evecs_normed.append(ev) 
1870          self.evecs = tuple(evecs_normed)

1871   


1872 -    def _ensure_jac(self, jac):


1873          if jac is None: 
1874              raise NotImplementedError('Currently, Jacobian must be supplied for nD') 
1875          else: 
1876              return jac

1877   


1878 -    def __getitem__(self, k):


1879          return self.point[k]

1880   


1881 -    def __setitem__(self, k, v):


1882          self.point[k] = v

1883   


1884 -    def _classify(self):


1885          if self.dimension == 2: 
1886              print "Use fixedpoint_2D class" 
1887          raise NotImplementedError("Not implemented for n dimensions where n != 2")

1888   
1889   


1890 -class fixedpoint_2D(fixedpoint_nD):


1891 -    def __init__(self, gen, pt, coords=None, jac=None, description='', normord=2, eps=1e-12):


1892          fixedpoint_nD.__init__(self, gen, pt, coords, jac, description=description, 
1893                                 normord=normord, eps=eps) 
1894          if self.dimension != 2: 
1895              raise TypeError("This class is for 2D systems only")

1896   


1897 -    def _ensure_jac(self, jac):


1898          # overrides nD versions 
1899          if jac is None: 
1900              try: 
1901                  J = self.gen.Jacobian(0, self.gen.initialconditions) 
1902              except PyDSTool_ExistError: 
1903                  # no full Jac available 
1904                  raise NotImplementedError('Jacobian not available') 
1905              else: 
1906                  # in the future, make a jac function that extracts 
1907                  # the n components from Jacobian (if n < gen.dimension) 
1908                  if J.shape[0] == J.shape[1] == self.dimension: 
1909                      def jac(t, x, y): 
1910                          return self.gen.Jacobian(t, {self.fp_coords[0]: x, 
1911                                                  self.fp_coords[1]: y})

1912                  else: 
1913                      raise NotImplementedError('Jacobian is not the right shape') 
1914          return jac

1915   


1916 -    def _classify(self):


1917          real_evals = (isreal(self.evals[0]), isreal(self.evals[1])) 
1918          equal_evals = abs(self.evals[0] - self.evals[1]) < self.eps 
1919          zero_evals = (abs(self.evals[0]) < self.eps, 
1920                        abs(self.evals[1]) < self.eps) 
1921          if alltrue(real_evals): 
1922              sign_evals = (sign(self.evals[0]), sign(self.evals[1])) 
1923              if sign_evals[0] == sign_evals[1]: 
1924                  self.classification = 'node' 
1925              else: 
1926                  self.classification = 'saddle' 
1927          else: 
1928              self.classification = 'spiral' 
1929          real_parts = real(self.evals) 
1930          if alltrue(real_parts<0): 
1931              self.stability = 's' 
1932          elif alltrue(real_parts==0): 
1933              self.stability = 'c' 
1934          else: 
1935              self.stability = 'u' 
1936          self.degenerate = sometrue(zero_evals) or equal_evals

1937   


1938 -    def toarray(self, restrict_to_coords=None):


1939          """restrict_to_coords option of ordered coordinate names (default None) 
1940          ensures the return array only contains values of those coordinates in 
1941          that order. Useful for interfacing with plotting commands.""" 
1942          if restrict_to_coords is None: 
1943              return self.point.coordarray 
1944          else: 
1945              ixs = self.point._map_names_to_ixs(restrict_to_coords) 
1946              return self.point.coordarray[ixs]

1947   
1948   


1949 -def make_distance_to_known_line_auxfn(fname, p, dp=None, q=None):


1950      """Builds definition of an auxiliary function of (x,y) measuring 
1951      (signed) distance from a 2D line given by the point p and either 
1952      (a) the vector dp, or (b) the point q. (All inputs must be Points.) 
1953      """ 
1954      if dp is None: 
1955          assert q is not None, "Only specify dp or q, not both" 
1956          assert len(p)==len(q)==2 
1957          other = q 
1958      else: 
1959          assert q is None, "Only specify dp or q, not both" 
1960          assert len(p)==len(dp)==2 
1961          other = dp 
1962      try: 
1963          assert p.coordnames == other.coordnames 
1964      except AttributeError: 
1965          raise TypeError("Only pass Point objects as inputs") 
1966      if dp is None: 
1967          q0_m_p0 = q[0]-p[0] 
1968          q1_m_p1 = q[1]-p[1] 
1969      else: 
1970          # based on idea that q - p = dp 
1971          # to prevent loss of precision 
1972          q0_m_p0 = dp[0] 
1973          q1_m_p1 = dp[1] 
1974      denom = sqrt(q0_m_p0*q0_m_p0 + q1_m_p1*q1_m_p1) 
1975      term = q0_m_p0*p[1]-q1_m_p1*p[0] 
1976      if denom == 1.0: 
1977          d = '%s-%s*y+%s*x'%(repr(term), repr(q0_m_p0), repr(q1_m_p1)) 
1978      else: 
1979          d = '(%s-%s*y+%s*x)/%s'%(repr(term), repr(q0_m_p0), 
1980                                   repr(q1_m_p1), repr(denom)) 
1981      return {fname: (['x','y'], d)}

1982   
1983   


1984 -def make_distance_to_line_auxfn(linename, fname, p, by_vector_dp=True):


1985      """Builds definition of an auxiliary function of (x,y) measuring 
1986      (signed) distance from a 2D line given at run-time by coordinate 
1987      names in the tuple of strings p and either a vector dp, 
1988      or a point q, depending on the second input argument. 
1989      Also returns list of parameter names used. 
1990      """ 
1991      assert len(p)==2 and isinstance(p[0], str) and isinstance(p[1], str) 
1992      p0 = linename+'_p_'+p[0] 
1993      p1 = linename+'_p_'+p[1] 
1994      pars = [p0, p1] 
1995      if by_vector_dp: 
1996          # based on idea that q - p = dp 
1997          # to prevent loss of precision 
1998          dp0 = linename+'_dp_'+p[0] 
1999          dp1 = linename+'_dp_'+p[1] 
2000          pars.extend([dp0, dp1]) 
2001          q0_m_p0 = dp0 
2002          q1_m_p1 = dp1 
2003      else: 
2004          q0 = linename+'_q_'+p[0] 
2005          q1 = linename+'_q_'+p[1] 
2006          pars.extend([q0, q1]) 
2007          q0_m_p0 = '(%s-%s)'%(q0,p0) 
2008          q1_m_p1 = '(%s-%s)'%(q1,p1) 
2009      denom = 'sqrt(%s*%s+%s*%s)'%(q0_m_p0,q0_m_p0,q1_m_p1,q1_m_p1) 
2010      p1_m_y = '(%s-y)'%(p1) 
2011      p0_m_x = '(%s-x)'%(p0) 
2012      d = '(%s*%s-%s*%s)/%s'%(q0_m_p0, p1_m_y, p0_m_x, q1_m_p1, denom) 
2013      return {'pars': pars, 'auxfn': {fname: (['x','y'], d)}}

2014   
2015   


2016 -def bisection(func, a, b, args=(), xtol=1e-10, maxiter=400, normord=2):


2017      """Bisection root-finding method.  Given a function returning +/- 1 
2018      and an interval with func(a) * func(b) < 0, find the root between a and b. 
2019   
2020      Variant of scipy.optimize.minpack.bisection with exception for too many iterations 
2021      """ 
2022      eva = func(a,*args) 
2023      evb = func(b,*args) 
2024      assert (eva*evb < 0), "Must start with interval with func(a) * func(b) <0" 
2025      i = 1 
2026      while i<=maxiter: 
2027          dist = (b-a)/2.0 
2028          p = a + dist 
2029          # in case dist is a scalar 
2030          if norm(np.asarray(dist).flatten(),normord) < xtol: 
2031              return p 
2032          try: 
2033              ev = func(p,*args) 
2034          except RuntimeError: 
2035              return p 
2036          if ev == 0: 
2037              return p 
2038          i += 1 
2039          if ev*eva > 0: 
2040              a = p 
2041              eva = ev 
2042          else: 
2043              b = p 
2044      raise RuntimeError("Method failed after %d iterations." % maxiter)

2045   
2046   
2047   
2048   
2049  # ------ 
2050   
2051   


2052 -class plotter_2D(object):


2053      """Plotting manager for phase plane analysis. A global instance of 
2054      this class called 'plotter' is already created and exported by this 
2055      module. Operate on that instance directly. 
2056   
2057      All plotting will take place in figure numbered self.curr_fig, which 
2058      will be resized to the given shape and limits. 
2059   
2060      All added methods of form plot_X take an optional named figure which would 
2061      correspond to whatever figure number is allocated in the fig_directory 
2062      dictionary attribute. New plot methods of this kind must first check 
2063      that do_display is True, and then call the 'setup' method before, and 
2064      'teardown' after doing the necessary plotting. 
2065   
2066      """ 


2067 -    def __init__(self, x_dom=None, y_dom=None, figsize=(6,5), 
2068                   curr_fig=1, do_display=False):


2069          self.x_dom = x_dom 
2070          self.y_dom = y_dom 
2071          self.do_display = do_display 
2072          self.figsize = figsize 
2073          self.curr_fig = 1 
2074          self.fig_directory = {} 
2075          # layers will permit types of plot data to be removed or restored 
2076          # to a given plot axes without recomputation 
2077          self.layers = {}

2078   


2079 -    def set_curr_fig(self, name):


2080          try: 
2081              self.curr_fig = self.fig_directory[name] 
2082          except KeyError: 
2083              raise ValueError("Create entry in fig_directory attribute for this figure name")

2084   


2085 -    def setup(self, figname):


2086          if figname is not None: 
2087              self.set_curr_fig(figname) 
2088          pp.figure(self.curr_fig, figsize=self.figsize)

2089   


2090 -    def teardown(self):


2091          pp.figure(self.curr_fig) 
2092          if self.x_dom is not None: 
2093              pp.xlim( self.x_dom ) 
2094          if self.y_dom is not None: 
2095              pp.ylim( self.y_dom ) 
2096          pp.draw()

2097   


2098 -    def plot_nullcline(self, nullc, style, lw=1, N=100, marker='', figname=None):


2099          if not self.do_display: 
2100              return 
2101          self.setup(figname) 
2102          x_data = nullc.array[:,0] 
2103          y_data = nullc.array[:,1] 
2104          xs = np.sort( np.concatenate( (np.linspace(x_data[0], x_data[-1], N), x_data) ) ) 
2105          ys = nullc.spline(xs) 
2106          pp.plot(xs, ys, style, linewidth=lw) 
2107          if marker != '': 
2108              pp.plot(x_data, y_data, style[0]+marker) 
2109          self.teardown()

2110   


2111 -    def plot_vf(self, gen, xname, yname, col='k', N=20, subdomain=None, scale_exp=0, 
2112                  figname=None):


2113          """Plot vector field in 2D""" 
2114          if not self.do_display: 
2115              return 
2116          self.setup(figname) 
2117          plot_PP_vf(gen, xname, yname, N=N, subdomain=subdomain, scale_exp=scale_exp) 
2118          self.teardown()

2119   


2120 -    def plot_fps(self, fps, coords=None, do_evecs=False, markersize=10, figname=None):


2121          """Plot fixed point(s) in 2D""" 
2122          if not self.do_display: 
2123              return 
2124          plot_PP_fps(fps, coords=coords, do_evecs=do_evecs, markersize=markersize) 
2125          self.teardown()

2126   


2127 -    def plot_line_from_points(self, A, B, style, lw=1, figname=None):


2128          if not self.do_display: 
2129              return 
2130          self.setup(figname) 
2131          pp.plot([A[0], B[0]], [A[1], B[1]], style, linewidth=lw) 
2132          self.teardown()

2133   


2134 -    def plot_point(self, A, style, figname=None):


2135          if not self.do_display: 
2136              return 
2137          self.setup(figname) 
2138          pp.plot(A[0], A[1], style) 
2139          self.teardown()

2140   


2141 -    def plot_vline(self, x, style, figname=None):


2142          if not self.do_display: 
2143              return 
2144          self.setup(figname) 
2145          pp.axvline(x, color=style[0], linestyle=style[1]) 
2146          self.teardown()

2147   


2148 -    def plot_hline(self, y, style, figname=None):


2149          if not self.do_display: 
2150              return 
2151          self.setup(figname) 
2152          pp.axhline(y=y, color=style[0], linestyle=style[1]) 
2153          self.teardown()

2154   
2155   
2156  # global instance 
2157  global plotter 
2158  plotter = plotter_2D() 
2159   


2160 -def _newton_step(Q0, nullc, A, B, phi, tol):


2161      """Internal function for line intersection involving splines. 
2162      Q0 is the initial point on the given nullcline. A closer approximation 
2163      Q on the nullcline where it intersects line AB""" 
2164      global plotter 
2165      # Still need to choose the 0.2 better to ensure correct scaling with A and B 
2166   
2167      err = 1000*tol 
2168      Q = Q0 
2169      while err > tol: 
2170          # Q0 prime is a distant point on the spline's tangent line at Q0 
2171          Q_prime = Q + 0.2*nullc.tgt_vec(Q.x) 
2172          #plotter.plot_line_from_points(Q, Q_prime, 'b') 
2173          P1 = line_intersection(Q, Q_prime, A, B) 
2174          Q1 = Point2D(P1.x, nullc.spline(P1.x))  # project onto spline 
2175          #plotter.plot_point(Q1, 'ko') 
2176          err = abs(phi-angle_to_vertical(Q1-A)) 
2177          Q = Q1 
2178      return Q

2179   
2180   


2181 -def find_nearest_sample_points_by_angle(thetas, phi, return_NaN=False):


2182      """Returns two successive indices of the angles in the array argument 
2183      thetas, for which theta values straddle angle phi. 
2184   
2185      This function is useful if phi represents the angle from the vertical of 
2186      the normal to a curve's tangent line, and thetas are angles from the 
2187      vertical of another curve in the plane. This function can therefore help 
2188      find closest or furthest perpendicular distances between curves. 
2189   
2190      Assumes thetas are in increasing order, and that phi is properly contained 
2191        between min(thetas) and max(thetas) (otherwise raises a ValueError). 
2192   
2193      For the corner case where one difference between angles is exactly 0 (not 
2194        generic), one of the thetas values given by the returned indices is 
2195        exactly phi.""" 
2196      global plotter 
2197      pos_diffs = (thetas - phi) > 0 
2198      if np.all(pos_diffs) or not np.any(pos_diffs): 
2199          if return_NaN: 
2200              return np.NaN 
2201          else: 
2202              raise ValueError("Outside domain") 
2203      else: 
2204          if pos_diffs[0]: 
2205              # Trues then Falses in pos_diffs 
2206              # first_neg_ix guaranteed not to be 0 
2207              first_neg_ix = np.argmin(np.asarray(pos_diffs, int)) 
2208              straddling_ixs = (first_neg_ix-1, first_neg_ix) 
2209          else: 
2210              # Falses then Trues in pos_diffs 
2211              first_pos_ix = np.argmax(np.asarray(pos_diffs, int)) 
2212              straddling_ixs = (first_pos_ix-1, first_pos_ix) 
2213          return straddling_ixs

2214   
2215   


2216 -def closest_perp_distance_between_sample_points(NullcA, NullcB, xa, x0B, x1B, 
2217                                                  newt_tol=1e-6):


2218      """Closest perpendicular distance from (xa, ya) on Nullcline A to Nullcline B, 
2219      given that it's known to be between sample points x0B and x1B on Nullcline B. 
2220   
2221      Uses a Newton-like step, solving up to an angular tolerance given by newt_tol. 
2222      """ 
2223      global plotter 
2224      ya = NullcA(xa) 
2225      a = np.array([xa,ya]) 
2226      tgt_vec_at_a = NullcA.tgt_vec(xa, rescale=True) 
2227      # normal has length 1 
2228      normal_at_a = make_vec_at_A_face_B(get_orthonormal(tgt_vec_at_a), 
2229                                  ya, NullcB(xa)) 
2230      #plotter.plot_line_from_points(a, a+tgt_vec_at_a, 'k:') 
2231      phi = angle_to_vertical(normal_at_a) 
2232   
2233      A = Point2D(a) 
2234      B = Point2D(a + 0.2*normal_at_a) 
2235      C = Point2D(x0B, NullcB(x0B)) 
2236      D = Point2D(x1B, NullcB(x1B)) 
2237   
2238      P0 = line_intersection(A, B, C, D) 
2239      Q0 = Point2D(P0.x, NullcB(P0.x))  # project onto spline 
2240      #theta0 = angle_to_vertical(Q0-a) 
2241   
2242      #plotter.plot_line_from_points(A, B, 'k-') 
2243      #plotter.plot_line_from_points(C, D, 'r--') 
2244      #plotter.plot_point(P0, 'gs') 
2245      #plotter.plot_point(Q0, 'ys') 
2246   
2247      try: 
2248          Q = _newton_step(Q0, NullcB, A, B, phi, newt_tol) 
2249      except ValueError: 
2250          return np.Inf 
2251      else: 
2252          vec = Q-A 
2253          #vec[0] = vec[0] / 100.  # hack to investigate differing x and y scales 
2254          return np.linalg.norm(vec)

2255   
2256   


2257 -def closest_perp_distance_on_spline(NullcA, NullcB, xa):


2258      """Closest perpendicular distance to Nullcline B at point given by xa on Nullcline A""" 
2259      global plotter 
2260      A = Point2D(xa, NullcA(xa)) 
2261      a_to_NullcB = NullcB.array - A 
2262      thetas_B = np.apply_along_axis(angle_to_vertical, -1, a_to_NullcB) 
2263      # monotonicity assumptions ensures that y coord of other nullcline 
2264      # indicates which direction normal vector must face 
2265      # ... also assumes that nullcline B is defined for all x values that 
2266      # nullcline A is 
2267      norm_vec_at_a = make_vec_at_A_face_B(get_orthonormal( \ 
2268                            NullcA.tgt_vec(xa, rescale=True)), 
2269                                           A.y, NullcB(xa)) 
2270      phi_a = angle_to_vertical(norm_vec_at_a) 
2271      #plotter.plot_line_from_points(A, A + norm_vec_at_a, 'k-.') 
2272   
2273      # determine closest sample points 
2274      try: 
2275          ix_B_lo, ix_B_hi = find_nearest_sample_points_by_angle(thetas_B, phi_a) 
2276      except ValueError: 
2277          # Outside domain of other nullcline 
2278          return np.Inf 
2279      else: 
2280          if ix_B_lo == ix_B_hi: 
2281              ix_B_lo = np.max( [0, ix_B_lo-1] ) 
2282              ix_B_hi = np.min( [len(NullcB.array)-1, ix_B_hi+1] ) 
2283          x0B = NullcB.array[ix_B_lo,0] 
2284          x1B = NullcB.array[ix_B_hi,0] 
2285   
2286      # refine to closest point on spline of Nullcline B 
2287      return closest_perp_distance_between_sample_points(NullcA, NullcB, xa, x0B, x1B)

2288   
2289   


2290 -def is_min_bracket(triple):


2291      """For a triple of positive numbers (a,b,c), returns a triple 
2292      (boolean, index, error) 
2293   
2294      where: 
2295      boolean is True if a > b < c (this brackets a minimum value); 
2296   
2297      index is 1 (middle) if input brackets a minimum value, otherwise 
2298      index indicates which end of the bracket has the smallest value; 
2299   
2300      error is the smallest absolute difference between distances in the 
2301      triple (a, b, c) 
2302      """ 
2303      a,b,c = triple 
2304      if b < a and b < c: 
2305          return True, 1, min( a-b, c-b ) 
2306      else: 
2307          if a < c: 
2308              # then monotonic a < b < c 
2309              return False, 0, min( b-a, c-b ) 
2310          else: 
2311              # a > c, monotonic a > b > c 
2312              return False, 2, min( a-b, b-c )

2313   
2314   


2315 -def _sample_array_interior(xdata, pc, refine=0):


2316      """xdata is a 1D array. 
2317      """ 
2318      # Consider moving into Interval class as a method... 
2319      all_xs = [] 
2320      if refine > 0: 
2321          dxs = xdata[1:] - xdata[:-1] 
2322          refine_dxs = 1/(1.0+refine) * dxs 
2323          for i, x in enumerate(xdata[:-1]): 
2324              all_xs.extend(list(x+refine_dxs[i]*arange(0, refine+1))) 
2325          all_xs.append(xdata[-1]) 
2326          xs = np.array(all_xs) 
2327      else: 
2328          xs = xdata.copy() 
2329      xs[0] += pc*(xs[1] - xs[0]) 
2330      xs[-1] -= pc*(xs[-1] - xs[-2]) 
2331      return xs

2332   
2333   


2334 -def closest_perp_distance_between_splines(NullcA, NullcB, dist_delta_tol=1e-5, 
2335                                            strict=True):


2336      """Measure closest perpendicular distance between two nullcline objects 
2337      (via their spline representations). 
2338   
2339      Tolerance argument measures that of Euclidean distance between the splines 
2340      representing the two nullclines. 
2341   
2342      strict option (default True) ensures both nullclines are monotonically 
2343      increasing or decreasing functions of x. If False, on nullcline A must be, 
2344      while nullcline B non-monotonicity will simply lead to a warning message 
2345      (since B plays an asymmetric role to A in the calculation, its monotonicity 
2346      is less critical). 
2347   
2348      Assumes monotonicity of both nullclines, which may be relaxed in future 
2349      versions. 
2350      """ 
2351      assert NullcA.is_monotonic(), "Nullcline A must be monotonic" 
2352      Bmon = NullcB.is_monotonic() 
2353      if strict: 
2354          assert Bmon, "Nullcline B must be monotonic" 
2355      else: 
2356          if not Bmon: 
2357              print "Warning: nullcline B is not monotonic in closest_perp_distance_between splines" 
2358      # search interior sample points of spline A first, and 1% inside endpoints 
2359      xa_search_vals = _sample_array_interior(NullcA.array[:,0], 0.01) 
2360      dists = [closest_perp_distance_on_spline(NullcA, NullcB, xa) for xa in xa_search_vals] 
2361   
2362      if isincreasing(dists): 
2363          xa_start_ix1 = 0 
2364          xa_start_ix2 = 1 
2365          xa_ix = 0 
2366      elif isincreasing(dists[::-1]): 
2367          xa_start_ix1 = len(dists)-2 
2368          xa_start_ix2 = len(dists)-1 
2369          xa_ix = len(dists)-1 
2370      else: 
2371          xa_ix = np.argmin(dists)  # used later too to recover associated xa value used 
2372          xa_start_ix1 = xa_ix ##+ 1 # ignored spline endpoint 
2373          other_dists = dists[:] 
2374          other_dists[xa_ix] = np.Inf 
2375          if np.any( np.isfinite( np.array(other_dists) )): 
2376              xa_start_ix2 = np.argmin(other_dists) ##+ 1 
2377              if xa_start_ix2 < xa_start_ix1: 
2378                  # switch 
2379                  xa_start_ix1, xa_start_ix2 = (xa_start_ix2, xa_start_ix1) 
2380          else: 
2381              # both ix1 and ix2 would be the same, so extend search over next 
2382              # neighboring indices, if available 
2383              xa_start_ix2 = min([len(dists), xa_start_ix1 + 1]) 
2384              xa_start_ix1 = max([0,xa_start_ix1 - 1]) 
2385   
2386      # Minimization method from xa over given indices in NullcA.array[ix1:ix2,0] 
2387      # except if either is an endpoint, in which case move in until distance to 
2388      # NullcB becomes well-defined 
2389      xa_lo = xa_search_vals[xa_start_ix1] 
2390      d_lo = dists[xa_start_ix1] 
2391      xa_hi = xa_search_vals[xa_start_ix2] 
2392      d_hi = dists[xa_start_ix2] 
2393   
2394      # Old code in case there was a problem proceeding using endpoints 
2395  ##    if xa_start_ix1 > 0: 
2396  ##        xa_lo = xa_search_vals[xa_start_ix1] 
2397  ##        d_lo = dists[xa_start_ix1] 
2398  ##    else: 
2399  ##        raise NotImplementedError 
2400  ## 
2401  ##    if xa_start_ix2 < len(NullcA.array): 
2402  ##        xa_hi = xa_search_vals[xa_start_ix2] 
2403  ##        d_hi = dists[xa_start_ix2] 
2404  ##    else: 
2405  ##        raise NotImplementedError 
2406   
2407      assert xa_hi > xa_lo 
2408   
2409      # given sufficient number of sample points for nullclines... 
2410      #   concavity test for NullcA: shortest dist either at endpoints (monotonic assumed), 
2411      #   or in interior (quadratic-like minimization). 
2412   
2413      xa_mid = xa_search_vals[xa_ix] # may be the same as one of the endpoints 
2414      if xa_mid in (xa_lo, xa_hi): 
2415          # xa_mid is the same as an endpoint 
2416          # split the difference midway 
2417          xa_mid = 0.5*(xa_lo+xa_hi) 
2418          d_mid = closest_perp_distance_on_spline(NullcA, NullcB, xa_mid) 
2419      else: 
2420          d_mid = dists[xa_ix] 
2421   
2422      # Begin bisection-like linearly-convergent minimization (with convergence 
2423      # guarantee due to bracketing) 
2424      x_bracket = (xa_lo, xa_mid, xa_hi) 
2425      d_bracket = (d_lo, d_mid, d_hi) 
2426   
2427      is_brack, ix_min, error = is_min_bracket(d_bracket) 
2428      #plotter.do_display = True 
2429   
2430      while error > dist_delta_tol: 
2431          a, b, c = x_bracket 
2432          if is_brack: 
2433              b_prime = 0.5*(a + b) # pick side to the "left" (w.l.o.g.) 
2434              d_new = closest_perp_distance_on_spline(NullcA, NullcB, b_prime) 
2435              # test bracket 1 is xa_lo, xa_new, xa_mid 
2436              test_bracket1 = (d_bracket[0], d_new, d_bracket[1]) 
2437              is_brack_test, ix_min, d_error = is_min_bracket(test_bracket1) 
2438              if is_brack_test: 
2439                  # done 
2440                  x_bracket = (a, b_prime, b) 
2441                  d_bracket = test_bracket1 
2442                  is_brack = True 
2443                  error = d_error 
2444                  continue # while 
2445              # test bracket 2 is xa_new, xa_mid, xa_hi 
2446              test_bracket2 = (d_new, d_bracket[1], d_bracket[2]) 
2447              is_brack_test, ix_min, d_error = is_min_bracket(test_bracket2) 
2448              if is_brack_test: 
2449                  # done 
2450                  x_bracket = (b_prime, b, c) 
2451                  d_bracket = test_bracket2 
2452                  is_brack = True 
2453                  error = d_error 
2454                  # continue while 
2455          else: 
2456              # true d function may be monotonic, but must try to find a better 
2457              # intermediate point that creates a bracket. 
2458   
2459              # assuming minimum exists for d in this range, 
2460              # pick a point between the two smallest d's and continue search for bracket. 
2461              if ix_min == 0: 
2462                  b_prime = 0.5*(a + b) 
2463                  d_new = closest_perp_distance_on_spline(NullcA, NullcB, b_prime) 
2464                  # does the new selection create a bracket? 
2465                  # test bracket 1 is xa_lo, xa_new, xa_mid 
2466                  d_bracket = (d_bracket[0], d_new, d_bracket[1]) 
2467                  x_bracket = (a, b_prime, b) 
2468              else: 
2469                  # ix_min == 2 
2470                  b_prime = 0.5*(b + c) 
2471                  d_new = closest_perp_distance_on_spline(NullcA, NullcB, b_prime) 
2472                  # does the new selection create a bracket? 
2473                  # test bracket 1 is xa_mid, xa_new, xa_hi 
2474                  d_bracket = (d_bracket[1], d_new, d_bracket[2]) 
2475                  x_bracket = (b, b_prime, c) 
2476              is_brack_test, ix_min, error = is_min_bracket(d_bracket) 
2477      return x_bracket[ix_min], d_bracket[ix_min]

2478   
2479   
2480  # EXPERIMENTAL CLASS 


2481 -class dx_scaled_2D(object):


2482      """Supports a delta x vector that automatically re-scales 
2483      according to the known scalings of each of the vector's component 
2484      directions. 
2485   
2486      Highly experimental class! 
2487      """ 
2488   


2489 -    def __init__(self, dx, scale=(1,1)):


2490          assert isreal(dx) # and dx>0 
2491          self.dx = dx 
2492          assert len(scale)==2 and scale[0] > 0 and scale[1] > 0 
2493          m = max(scale) 
2494          self.x_scale = scale[0]/m 
2495          self.y_scale = scale[1]*1./scale[0]/m

2496   


2497 -    def __call__(self, dir):


2498          # angle is between 0 and 1: 0 = entirely in x-axis, 1 = entirely in y-axis 
2499          # angle gives relative y-axis contribution of dir vector 
2500          try: 
2501              angle = 2*atan2(dir[1],dir[0])/pi 
2502          except TypeError: 
2503              # e.g. unsubscriptable object, from a __mul__ call 
2504              return self.dx*dir 
2505          else: 
2506              return self.dx*dir*(angle*self.y_scale+(1-angle))

2507   
2508      __mul__ = __call__ 
2509   


2510 -    def __rmul__(self, dir):


2511          # angle is between 0 and 1: 0 = entirely in x-axis, 1 = entirely in y-axis 
2512          # angle gives relative y-axis contribution of dir vector 
2513          try: 
2514              angle = 2*atan2(dir[1],dir[0])/pi 
2515          except TypeError: 
2516              # e.g. unsubscriptable object 
2517              return dx_scaled_2D(self.dx*dir, (1,self.y_scale)) 
2518          else: 
2519              return self.dx*dir*(angle*self.y_scale+(1-angle))

2520   
2521   


2522 -def find_saddle_manifolds(fp, dx=None, dx_gamma=None, dx_perp=None, tmax=None, 
2523                            max_len=None, ic=None, 
2524                            ic_dx=None, max_pts=1000, directions=(1,-1), 
2525                            which=('s', 'u'), other_pts=None, rel_scale=None, 
2526                            dx_perp_fac=0.75, verboselevel=0, fignum=None):


2527      """Compute any branch of the stable or unstable sub-manifolds of a saddle. 
2528      Accepts fixed point instances of class fixedpoint_2D. 
2529   
2530      Required inputs: 
2531        fp:       fixed point object 
2532        dx:       arc-length step size (**fixed**) 
2533        dx_gamma: determines the positions of the Gamma_plus and Gamma_minus 
2534                  event surfaces (can be a real scalar or a pair if not symmetric) 
2535        dx_perp:  initial perturbation from the local linear sub-manifolds to 
2536                  find starting points. 
2537        tmax:     maximum time to compute a trajectory before 'failing' 
2538        max_len:  maximum arc length to compute 
2539        max_pts:  maximum number of points to compute on each sub-manifold branch 
2540        Specify either ic or ic_dx for initial point (e.g. to restart the calc 
2541          after a previous failure) or a certain distance from the saddle point. 
2542   
2543      Optional inputs: 
2544        rel_scale:  a pair giving relative scalings of x and y coordinates in 
2545          the plane, to improve stepping in the different directions. 
2546          e.g. (1,10) would make dx steps in the y-direction 10 times larger than 
2547          in the x-direction. 
2548   
2549        which:  which sub-manifold to compute 's', 'u' or ('s', 'u'). 
2550          Default is both. 
2551   
2552        directions:  which directions along chosen sub-manifolds? (1,), (-1,) 
2553          or (1,-1). Default is both. 
2554   
2555        other_pts can be a list of points whose proximity will be checked, 
2556      and the computation halted if they get within dx of the manifold. 
2557   
2558        dx_perp_fac:  For advanced use only. If you get failures saying dx_perp 
2559           too small and that initial displacement did not straddle manifold, try 
2560           increasing this factor towards 1 (default 0.75). Especially for 
2561           unstable manifolds, initial values for dx_perp may diverge, but if 
2562           dx_perp is shrunk too quickly with this factor the sweet spot may be 
2563           missed. 
2564   
2565        verboselevel 
2566        fignum 
2567      """ 
2568   
2569      assert fp.classification == 'saddle' and not fp.degenerate 
2570      if fp.evals[0] < 0: 
2571          eval_s = fp.evals[0] 
2572          eval_u = fp.evals[1] 
2573          evec_s = fp.evecs[0] 
2574          evec_u = fp.evecs[1] 
2575      else: 
2576          eval_s = fp.evals[1] 
2577          eval_u = fp.evals[0] 
2578          evec_s = fp.evecs[1] 
2579          evec_u = fp.evecs[0] 
2580      gen = fp.gen 
2581      assert 'Gamma_out_plus' in gen.eventstruct, "Detection event surface(s) not present" 
2582      assert 'Gamma_out_minus' in gen.eventstruct, "Detection event surface(s) not present" 
2583      # Dividing fixed point's inherited epsilon tolerance by 100 
2584      eps = fp.eps / 100 
2585      dx_perp_eps = 1e-12 
2586      if dx_perp_fac >= 1 or dx_perp_fac <= 0: 
2587          raise ValueError("dx_perp_fac must be between 0 and 1") 
2588      normord = fp.normord 
2589      if rel_scale is None: 
2590          rel_scale = (1,1) 
2591      dxscaled = dx_scaled_2D(dx, rel_scale) 
2592      if isinstance(dx_gamma, dict): 
2593          assert len(dx_gamma) == 2, "Invalid value for dx_gamma" 
2594          assert remain(dx_gamma.keys(), [1,-1]) == [], \ 
2595              "Invalid value for dx_gamma" 
2596      else: 
2597          try: 
2598              dx_gamma = {1: dx_gamma, -1: dx_gamma} 
2599          except: 
2600              raise TypeError("Invalid type for dx_gamma") 
2601   
2602      def test_fn(x, dircode): 
2603          if verboselevel>1: 
2604              print "Test point", x[x.coordnames[0]], x[x.coordnames[1]], "in direction", dircode, "\n" 
2605          gen.set(ics=x) 
2606          try: 
2607              test = gen.compute('test', dirn=dircode) 
2608          except: 
2609              raise RuntimeError("Integration failed") 
2610          events = gen.getEvents() 
2611          if verboselevel>1: 
2612              pts=test.sample(coords=x.coordnames) 
2613              plot(pts[x.coordnames[0]][:25],pts[x.coordnames[1]][:25],'b-') 
2614          if events['Gamma_out_plus'] is None: 
2615              if events['Gamma_out_minus'] is None: 
2616                  if verboselevel>1: 
2617                      pts=test.sample(coords=x.coordnames) 
2618                      print "Last computed point was\n", pts[-1] 
2619                      print "...after time", pts['t'][-1] 
2620                      plot(pts[x.coordnames[0]],pts[x.coordnames[1]],'b-') 
2621                  raise RuntimeError("Did not reach Gamma surfaces") 
2622              else: 
2623                  # hit Gamma_out_minus 
2624                  if verboselevel>1: 
2625                      print "Reached Gamma minus at t=", events['Gamma_out_minus']['t'][0] 
2626                  sgn = -1 
2627          else: 
2628              if events['Gamma_out_minus'] is None: 
2629                  # hit Gamma_out_plus 
2630                  if verboselevel>1: 
2631                      print "Reached Gamma plus at t=", events['Gamma_out_plus']['t'][0] 
2632                  sgn = 1 
2633              else: 
2634                  if verboselevel>1: 
2635                      pts=test.sample(coords=x.coordnames) 
2636                      print "Last computed point was\n", pts[-1] 
2637                      print "...after time", pts['t'][-1] 
2638                      plot(pts[x.coordnames[0]],pts[x.coordnames[1]],'b-') 
2639                  raise RuntimeError("Did not reach Gamma surfaces") 
2640          return sgn

2641   
2642      def onto_manifold(x_ic, dn, normal_dir, dircode='f'): 
2643          try: 
2644              return bisection(test_fn, x_ic+dn*normal_dir, x_ic-dn*normal_dir, 
2645                              args=(dircode,), xtol=eps, maxiter=100, 
2646                              normord=normord) 
2647          except AssertionError: 
2648              if verboselevel>1: 
2649                  xp = x_ic+dn*normal_dir 
2650                  xm = x_ic-dn*normal_dir 
2651                  plot(xp[var_x], xp[var_y], 'gx') 
2652                  plot(xm[var_x], xm[var_y], 'gx') 
2653              raise RuntimeError("dx_perp too small? +/- initial displacement did not straddle manifold") 
2654          except RuntimeError: 
2655              if verboselevel>1: 
2656                  xp = x_ic+dn*normal_dir 
2657                  xm = x_ic-dn*normal_dir 
2658                  plot(xp[var_x], xp[var_y], 'gx') 
2659                  plot(xm[var_x], xm[var_y], 'gx') 
2660              raise 
2661   
2662      gen.eventstruct['Gamma_out_plus'].activeFlag=True  # terminal 
2663      gen.eventstruct['Gamma_out_minus'].activeFlag=True  # terminal 
2664      var_x = fp.point.coordnames[0] 
2665      var_y = fp.point.coordnames[1] 
2666      assert tmax > 0 
2667      manifold = {} 
2668      man_names = {'s': 'stable', 'u': 'unstable'} 
2669   
2670      for w in which: 
2671          ### w = 's' => stable branch 
2672          ### w = 'u' => unstable branch 
2673          if verboselevel>0: 
2674              print "Starting %s branch" % man_names[w] 
2675          if w == 's': 
2676              col = 'g' 
2677              w_sgn = -1 
2678              integ_dircode = 'f' 
2679              evec = evec_u 
2680              evec_other = evec_s 
2681          elif w == 'u': 
2682              col = 'r' 
2683              w_sgn = 1 
2684              integ_dircode = 'b' 
2685              evec = evec_s 
2686              evec_other = evec_u 
2687          # set Gamma_out surfaces on "outgoing" branch 
2688          # (polarity is arbitrary) 
2689          p0_plus = fp.point + dx_gamma[1]*evec 
2690          p0_minus = fp.point - dx_gamma[-1]*evec 
2691          evec_perp = get_perp(evec) 
2692          # !! Must choose the event directions correctly 
2693          # Algorithm should be based on: event dircode = 1 
2694          # if ev(traj(ev_t - delta)) < 0 and ev(traj(ev_t + delta)) > 0 
2695          # where the evec along the flow towards the event surfaces 
2696          # determines which is "before" and which is "after" the 
2697          # event surface (time may be reversed depending on which 
2698          # manifold is being computed) 
2699          print "Set these event directions according to your problem..." 
2700          gen.eventstruct.setEventDir('Gamma_out_plus', -1) 
2701          gen.eventstruct.setEventDir('Gamma_out_minus', 1) 
2702          gen.set(pars={'Gamma_out_plus_p_'+var_x: p0_plus[var_x], 
2703                        'Gamma_out_plus_p_'+var_y: p0_plus[var_y], 
2704                        'Gamma_out_plus_dp_'+var_x: evec_perp[var_x], 
2705                        'Gamma_out_plus_dp_'+var_y: evec_perp[var_y], 
2706                        'Gamma_out_minus_p_'+var_x: p0_minus[var_x], 
2707                        'Gamma_out_minus_p_'+var_y: p0_minus[var_y], 
2708                        'Gamma_out_minus_dp_'+var_x: evec_perp[var_x], 
2709                        'Gamma_out_minus_dp_'+var_y: evec_perp[var_y], 
2710      ##                  'fp_'+var_x: fp.point[var_x], 'fp_'+var_y: fp.point[var_y] 
2711                        }, 
2712                  tdata = [0,tmax]) 
2713          if verboselevel>1: 
2714              if fignum is None: 
2715                  fignum=figure() 
2716              else: 
2717                  figure(fignum) 
2718              plot([p0_plus[var_x]-dxscaled*evec_perp[var_x],p0_plus[var_x]+dxscaled*evec_perp[var_x]], 
2719                   [p0_plus[var_y]-dxscaled*evec_perp[var_y],p0_plus[var_y]+dxscaled*evec_perp[var_y]], 'k-', linewidth=2) 
2720              plot([p0_minus[var_x]-dxscaled*evec_perp[var_x],p0_minus[var_x]+dxscaled*evec_perp[var_x]], 
2721                   [p0_minus[var_y]-dxscaled*evec_perp[var_y],p0_minus[var_y]+dxscaled*evec_perp[var_y]], 'k-', linewidth=2) 
2722              draw() 
2723          check_other_pts = other_pts is not None 
2724          piece = {} 
2725          if ic_dx is None: 
2726              ic_dx = dxscaled 
2727          else: 
2728              ic_dx = dx_scaled_2D(ic_dx, rel_scale) 
2729          if ic is None: 
2730              ic = fp.point 
2731              f_ic = -w_sgn * evec_other 
2732              curve_len = 0 
2733              # initial estimate x0 = a point close to f.p. along manifold with 
2734              # opposite stability 
2735          else: 
2736              # initial curve length from previous independent variable, if present 
2737              # otherwise, assume zero 
2738              if isinstance(ic, Pointset): 
2739                  assert len(ic) == 1, "Only pass a length-1 pointset" 
2740                  # guarantee curve_len > 0 
2741                  curve_len = abs(ic['arc_len'][0]) 
2742                  ic = ic[0] 
2743              else: 
2744                  curve_len = 0 
2745              f_ic = -w_sgn * gen.Rhs(0, ic, gen.pars)  # array 
2746          for sgn in directions: 
2747              if verboselevel>0: 
2748                  print "Starting direction", sgn 
2749              # PREDICTION 
2750              x0_ic = ic+w_sgn*sgn*ic_dx*f_ic/norm(f_ic, normord) 
2751              if verboselevel>1: 
2752                  figure(fignum) 
2753                  plot(x0_ic[var_x], x0_ic[var_y], 'go', linewidth=1) 
2754              # put x0 initial estimate onto stable manifold 
2755              f = -w_sgn * gen.Rhs(0, x0_ic, gen.pars)  # array 
2756              norm_to_flow = get_perp(f/norm(f, normord)) 
2757              if verboselevel>1: 
2758                  plot([x0_ic[var_x], x0_ic[var_x]+dxscaled*f[0]/norm(f,normord)], 
2759                       [x0_ic[var_y], x0_ic[var_y]+dxscaled*f[1]/norm(f,normord)], 
2760                       'r-') 
2761                  plot([x0_ic[var_x], x0_ic[var_x]+dxscaled*norm_to_flow[0]], 
2762                       [x0_ic[var_y], x0_ic[var_y]+dxscaled*norm_to_flow[1]], 
2763                       'r:') 
2764              dx_perp_default = dx_perp 
2765              # CORRECTION 
2766              while dx_perp > dx_perp_eps: 
2767                  try: 
2768                      x = onto_manifold(x0_ic, dx_perp, norm_to_flow, 
2769                                        dircode=integ_dircode) 
2770                  except RuntimeError, e: 
2771                      dx_perp *= dx_perp_fac 
2772                  else: 
2773                      break 
2774              if dx_perp <= dx_perp_eps: 
2775                  # RuntimeError was raised and could not continue reducing dx_perp 
2776                  print "dx_perp reached lower tolerance =", dx_perp_eps 
2777                  print e 
2778                  raise RuntimeError("Initial point did not converge") 
2779              else: 
2780                  curve_len += norm(x-ic, normord) 
2781                  piece[sgn*curve_len] = x 
2782                  num_pts = 1 
2783                  last_x = x 
2784                  if verboselevel>0: 
2785                      print "Initial point converged to (%.6f, %.6f)\n" % \ 
2786                            (x[var_x], x[var_y]) 
2787              dx_perp = dx_perp_default 
2788              last_f = f_ic 
2789              # step backwards along flow 
2790              while curve_len < max_len and num_pts < max_pts: 
2791                  if verboselevel>0: 
2792                      figure(fignum) 
2793                      plot(last_x[var_x], last_x[var_y], col+'.', linewidth=1) 
2794                  if check_other_pts and sometrue([norm(last_x - pt, normord) < dx \ 
2795                                   for pt in other_pts]): 
2796                      # we've hit a different fixed point (or other feature), so stop 
2797                      break 
2798                  f = -w_sgn * gen.Rhs(0, last_x, gen.pars) 
2799                  if all(sign(f) != sign(last_f)): 
2800                      f = -f 
2801                      # on other side of manifold so must keep stepping in the 
2802                      # same direction, therefore switch signs! 
2803                  # PREDICTION 
2804                  x_ic = last_x + w_sgn*sgn*dxscaled*f/norm(f,normord) 
2805                  last_f = f 
2806                  if verboselevel>1: 
2807                      print "\nStarting from point ", last_x 
2808                      delta = w_sgn*sgn*dxscaled*f/norm(f,normord) 
2809                      print "Trying point ", x_ic, "in direction (%.6f, %.6f)\n" % (delta[0], delta[1]) 
2810                  dx_perp = dx_perp_default 
2811                  # CORRECTION 
2812                  while dx_perp > dx_perp_eps: 
2813                      try: 
2814                          x = onto_manifold(x_ic, dx_perp, get_perp(f/norm(f,normord)), 
2815                                            dircode=integ_dircode) 
2816                      except RuntimeError, e: 
2817                          dx_perp *= 0.75 
2818                      else: 
2819                          break 
2820                  if dx_perp <= dx_perp_eps: 
2821                      # RuntimeError was raised and could not continue reducing dx_perp 
2822                      print "dx_perp reached lower tolerance =", dx_perp_eps 
2823                      print e 
2824                      break  # end while search 
2825                  else: 
2826                      curve_len += norm(x-last_x, normord) 
2827                      piece[sgn*curve_len] = x 
2828                      last_x = x 
2829                      num_pts += 1 
2830                      if verboselevel>1: 
2831                          print "\nManifold has %i points" % num_pts 
2832                      elif verboselevel>0: 
2833                          print ".", 
2834                          sys.stdout.flush() 
2835          if verboselevel>0: 
2836              # finish the line 
2837              print " " 
2838          indepvar, piece_sorted = sortedDictLists(piece, byvalue=False) 
2839          manifold[w] = pointsToPointset(piece_sorted, indepvarname='arc_len', 
2840                                         indepvararray=indepvar, norm=normord) 
2841      gen.eventstruct['Gamma_out_plus'].activeFlag=False 
2842      gen.eventstruct['Gamma_out_minus'].activeFlag=False 
2843  ##    gen.eventstruct['fp_closest'].activeFlag=False 
2844   
2845      return manifold 
2846   
2847   


2848 -def make_flow_normal_event(x, y, dxdt, dydt, targetlang, flatspec=None, 
2849                             fnspec=None, evtArgs=None):


2850      """For 2D vector fields only. 
2851   
2852      Supply flatspec if built vector field using ModelConstructor tools, 
2853      otherwise specify funcspec argument. 
2854      """ 
2855      x_par = x+'_normflow_p' 
2856      y_par = y+'_normflow_p' 
2857   
2858      if fnspec is None: 
2859          if flatspec is None: 
2860              raise ValueError("Must supply one of funcspec or flatspec") 
2861          varnames=[] 
2862          parnames=[] 
2863          inputnames=[] 
2864          fnspecs={} 
2865      else: 
2866          if flatspec is not None: 
2867              raise ValueError("Must supply only one of funcspec or flatspec") 
2868          try: 
2869              varnames = fnspec['vars'] 
2870          except KeyError: 
2871              varnames = [] 
2872          try: 
2873              parnames = fnspec['pars'] 
2874          except KeyError: 
2875              parnames = [] 
2876          try: 
2877              inputnames = fnspec['inputs'] 
2878          except KeyError: 
2879              inputnames = [] 
2880          try: 
2881              fnspecs = fnspec['auxfns'] 
2882          except KeyError: 
2883              fnspecs = {} 
2884      if evtArgs is None: 
2885          evtArgs = {'name': 'flow_normal_2D_evt', 
2886                     'eventtol': 1e-5, 
2887                     'eventdelay': 1e-4, 
2888                     'starttime': 0, 
2889                     'precise': True, 
2890                     'term': False} 
2891      else: 
2892          evtArgs['term'] = False 
2893          evtArgs['name'] = 'flow_normal_2D_evt' 
2894      v_dot_f = '(' + x_par + ' - ' + x + ') * (' + dxdt + ') + ' + \ 
2895                '(' + y_par + ' - ' + y + ') * (' + dydt + ')' 
2896      norm_v = 'sqrt(' + x_par + '*' + x_par + '+' + y_par + '*' + y_par + ')' 
2897      norm_f = 'sqrt(pow((' + dydt + '),2) + pow((' + dxdt + '),2))' 
2898      flow_n_str = v_dot_f + '/(' + norm_v + '+' + norm_f + ')' 
2899      ev = Events.makeZeroCrossEvent(expr=flow_n_str, 
2900                                   dircode=0, 
2901                                   argDict=evtArgs, 
2902                                   targetlang=targetlang, 
2903                                   varnames=varnames, 
2904                                   parnames=parnames+[x_par,y_par], 
2905                                   inputnames=inputnames, fnspecs=fnspecs, 
2906                                   flatspec=flatspec) 
2907      ev_helper = {'event_names': {2:'flow_normal_2D_evt'}, # 2 for 2D 
2908                   'pars_to_vars': {x_par:x, y_par:y}} 
2909      return (ev, ev_helper)

2910   
2911   


2912 -class phaseplane(object):


2913      """Working environment for 2D phase-plane analysis. 
2914   
2915      Effectively a thin wrapper around a Generator class 
2916      (will later be around a Model class, in order to support hybrid 
2917      2D vector fields) 
2918      """ 
2919   


2920 -    def __init__(self, vf_info, x_axis='', 
2921                   name='', normord=2, eps=1e-12):


2922          """Use x_axis to specify which variable should appear on the x-axis""" 
2923   
2924          assert isreal(eps) and eps > 0, "eps tolerance must be a positive real number" 
2925          self.eps = eps 
2926          assert isinstance(normord, int) and normord > 0, "normord must be a positive integer" 
2927          self.normord = normord 
2928          self.name = name 
2929          try: 
2930              assert isinstance(vf_info.ODEclass, ODEsystem), \ 
2931                 "Can only use this class with ODE system Generators" 
2932              # Should make this compatible with ModelSpec construction too 
2933              assert len(vf_info.ODEargs.vars)==2, "Only 2D systems permitted for phase planes" 
2934          except AttributeError: 
2935              raise TypeError("Invalid form given for vf_info") 
2936          self.vf_info = vf_info 
2937          # store copy of the actual vector definition to make sure this doesn't 
2938          # change when things are added or removed and the vf is rebuilt -- 
2939          # otherwise all self.objects will be invalidated 
2940          self._vf_core_copy = copy.copy(vf_info.ODEargs) 
2941          if x_axis != '': 
2942              assert x_axis in vf_info.ODEargs.vars 
2943              self.x_axis = x_axis 
2944              self.y_axis = remain(vf_info.ODEargs.vars, x_axis)[0] 
2945          else: 
2946              self.x_axis, self.y_axis = vf_info.ODEargs.vars 
2947          try: 
2948              self.build_vf() 
2949          except ValueError: 
2950              self.vf = None 
2951          self.objects = args(fixedpoints=None,nullclines=None,limitcycles=None, 
2952                              submanifolds=None,trajectories=None,specialpoints=None)

2953   


2954 -    def build_vf(self):


2955          # Later, wrap generator into a Model object using embed() 
2956          # Verify that self._vf_info.ODEargs has same core attributes as 
2957          # self._vf_core_copy, otherwise raise an exception 
2958          try: 
2959              self.vf = self.vf_info.ODEclass.__new__(self.vf_info.ODEclass, 
2960                                                      self.vf_info.ODEargs) 
2961          except: 
2962              raise ValueError("Invalid or incomplete parameters for building vector field Generator")

2963   


2964 -    def add(self, attribute, value):


2965          try: 
2966              setattr(self.vf_info['args'], attribute, value) 
2967          except: 
2968              raise ValueError("Invalid vector field attribute %s or value %s"%(attribute,str(value)))

2969   


2970 -    def remove(self, attribute, value):


2971          try: 
2972              del self.vf_info['args'][attribute][value] 
2973          except: 
2974              raise ValueError("Could not delete vector field value %s of attribute %s"%(str(value), attribute))

2975   
2976   
2977   


2978 -def find_period(pts, thresh, dir=1, with_indices=False):


2979      """pts is a Pointset. 
2980   
2981      thresh is a 1D Point or a dictionary, whose coordinate name corresponds 
2982      to a variable in the pts pointset. 
2983   
2984      dir is either 1 or -1, indicating which direction the threshold must be 
2985      crossed to be counted (default 1 = increasing). 
2986   
2987      with_indices is a Boolean indicating whether to return the pair of indices 
2988      indicating the beginning and end points of the last period (default False). 
2989      """ 
2990      try: 
2991          threshdict = dict(thresh) 
2992      except: 
2993          raise TypeError("thresh must be a 1D Point object or dictionary") 
2994      assert len(threshdict)==1, "thresh must be a 1D Point object or dictionary" 
2995      var = thresh.keys()[0] 
2996      a = pts[var] 
2997      t = pts['t'] 
2998      ts = [] 
2999      ixs = [] 
3000      th = thresh[var] 
3001      # if don't start on appropriate side of thresh, wait 
3002      def inc_fn(x): 
3003          return x < th

3004      def dec_fn(x): 
3005          return x > th 
3006      if dir == 1: 
3007          off_fn = inc_fn 
3008      elif dir == -1: 
3009          off_fn = dec_fn 
3010      else: 
3011          raise ValueError("dir must be 1 or -1") 
3012      off = off_fn(a[0]) 
3013      for i,av in enumerate(a): 
3014          if off and not off_fn(av): 
3015              ts.append(t[i]) 
3016              ixs.append(i) 
3017              off = False 
3018  ##            print "Detected at i=%d, a=%f"%(i,av) 
3019  ##            print ts 
3020          elif not off and off_fn(av): 
3021              off = True 
3022      if len(ts) >= 1: 
3023          if with_indices: 
3024              return ts[-1]-ts[-2], ixs 
3025          else: 
3026              return ts[-1]-ts[-2] 
3027      else: 
3028          print len(ts), "is not enough periods", 
3029          return NaN 
3030   
3031   
3032  # --------------------------------------------------------------- 
3033   


3034 -def _filter_consecutive(indices, minimize_values=None):


3035      """Return index array containing no consecutive values. 
3036      if minimize_values option is given, this sequence must contain positions for 
3037      all indices in the first argument, and will be used to choose which of any 
3038      consecutive indices to retain in the returned sequence. Otherwise, the first 
3039      index will be chosen arbitrarily. 
3040   
3041      E.g. to find the *last* index of every cluster instead of the default, use 
3042        minimize_values=range(max_index,0,-1) 
3043      """ 
3044      ## Find clusters 
3045      clusters = [map(operator.itemgetter(1), g) for k, g in \ 
3046                  itertools.groupby(enumerate(indices), lambda (i,x):i-x)] 
3047      ## Minimize on clusters 
3048      result = [] 
3049      for c in clusters: 
3050          if len(c) == 1: 
3051              result.append(c[0]) 
3052          else: 
3053              if minimize_values is None: 
3054                  result.append( c[0] ) 
3055              else: 
3056                  mvals = np.array(minimize_values) 
3057                  representative = np.argmin(np.array([mvals[c]])) 
3058                  result.append( c[representative] ) 
3059      return np.array(result)

3060   
3061   
3062  # Feature sub-classes 


3063 -class zone_node(ql_feature_node):


3064      """Phase plane 'zone' node of hierarchical qualitative feature abstract class. 
3065      A zone is a bounding box in the phase plane containing a feature, grown to include 
3066      a region that satisfies some local condition. 
3067   
3068      Must define _leaf_class for each implementation of a zone_node class 
3069      """ 


3070 -    def finish(self, target):


3071          self.subfeatures = dict.fromkeys(self.results.zone_center_ixs, None) 
3072          for ix in self.results.zone_center_ixs: 
3073              feat = self._leaf_class('zone_%i'%ix, pars=self.pars) 
3074              feat.pars.all_zone_ixs = list(self.results.zone_center_ixs) 
3075              feat.pars.index = ix 
3076              self.subfeatures[ix] = feat 
3077              feat(target)

3078   


3079 -    def _local_init(self):


3080          if not hasattr(self.pars, 'refine'): 
3081              self.pars.refine = 0

3082   
3083   


3084 -class zone_leaf(ql_feature_leaf):


3085      """Phase plane 'zone' for leaf of hierarchical qualitative feature abstract class. 
3086      A zone is a bounding box in the phase plane containing a feature, grown to include 
3087      a region that satisfies some local condition. 
3088      """ 


3089 -    def evaluate(self, target):


3090          self.results.zones = None

3091   


3092 -    def finish(self, target):


3093          """Override this function to be called if evaluate returns True""" 
3094          pass

3095   
3096   
3097  # Abstract classes 


3098 -class nullcline_zone_node(zone_node):


3099      pass

3100   
3101   


3102 -class nullcline_zone_leaf(zone_leaf):


3103      """Parameters to apply: 
3104      pars: xtol, refine (integer, default 0) 
3105      optional pars: find_exact_center (unused) 
3106      """ 


3107 -    def _prepare(self, nullc):


3108          center_ix = self.pars.index 
3109          x_center_approx, y_center_approx = nullc.array[center_ix] 
3110          # Do we care what is the exact position of the zone's defining position? 
3111          try: 
3112              exact = self.pars.find_exact_center 
3113          except AttributeError: 
3114              # default False 
3115              exact = False 
3116          if exact: 
3117              raise NotImplementedError 
3118          else: 
3119              x_center = x_center_approx 
3120              y_center = y_center_approx 
3121          return center_ix, x_center, y_center

3122   


3123 -    def _set_mutex_zone_by_width(self, center_ix, x_center, y_center, nullc):


3124          """Fix zone size by width or halfway to next center, if closer.""" 
3125          try: 
3126              zone_width = self.pars.zone_width 
3127          except AttributeError: 
3128              raise ValueError("Must set a zone_width parameter for this feature") 
3129          min_zone_x = x_center - zone_width 
3130          max_zone_x = x_center + zone_width 
3131          x_range = [np.nan, np.nan] 
3132          y_range = [np.nan, np.nan] 
3133          zone_position = self.pars.all_zone_ixs.index(center_ix) 
3134          if zone_position == 0: 
3135              # everything to the left is already included 
3136              x_range[0] = max( nullc.array[0,0], min_zone_x ) 
3137          else: 
3138              ix = self.pars.all_zone_ixs[zone_position-1] 
3139              x_range[0] = max( 0.5*(nullc.array[ix,0]+x_center), min_zone_x ) 
3140          y_range[0] = nullc(x_range[0]) 
3141   
3142          if center_ix == self.pars.all_zone_ixs[-1]: 
3143              # everything to the right is already included 
3144              x_range[1] = min( nullc.array[-1,0], max_zone_x ) 
3145          else: 
3146              ix = self.pars.all_zone_ixs[zone_position+1] 
3147              x_range[1] = min( 0.5*(nullc.array[ix,0]+x_center), max_zone_x ) 
3148          y_range[1] = nullc(x_range[1]) 
3149          return [np.array(x_range), np.array(y_range)]

3150   
3151   


3152 -    def _grow_zone_by_radius(self, center_ix, x_center, y_center, nullc, property_func):


3153          raise NotImplementedError

3154   


3155 -    def _grow_zone_by_width(self, center_ix, x_center, y_center, nullc, property_func):


3156          """property_func must return +/- 1 or 0/1 as a function of x""" 
3157          try: 
3158              zone_width = self.pars.zone_width 
3159          except AttributeError: 
3160              zone_width = np.inf  # may fail unexpectedly 
3161              print "Warning: zone_width parameter defaulted to infinity" 
3162          min_zone_x = x_center - zone_width 
3163          max_zone_x = x_center + zone_width 
3164          # Grow domain of zone past known sample points with same property 
3165          # until intermediate position between sample points found 
3166          x_range = [np.nan, np.nan] 
3167          y_range = [np.nan, np.nan] 
3168          zone_position = self.pars.all_zone_ixs.index(center_ix) 
3169   
3170          # Left search 
3171          if zone_position == 0: 
3172              # everything to the left is already included 
3173              x_range[0] = max( nullc.array[0,0], min_zone_x ) 
3174              y_range[0] = nullc(x_range[0]) 
3175          else: 
3176              # all sample points with indices ix_after:center_ix (inclusive) 
3177              # have the same property. 
3178              ix_before = self.pars.all_zone_ixs[zone_position-1] 
3179              ix_after = ix_before + 1 
3180              if nullc.array[ix_after,0] < min_zone_x: 
3181                  x_range[0] = min_zone_x 
3182                  y_range[0] = nullc(x_range[0]) 
3183              else: 
3184                  # search between these points on the nullcline 
3185                  xsol = bisection(property_func, nullc.array[ix_before,0], 
3186                                    nullc.array[ix_after,0], xtol=self.pars.xtol) 
3187                  if abs(xsol - nullc.array[ix_before,0]) < self.pars.xtol: 
3188                      # corner case where zero is right at a sample point 
3189                      # try again with extended sample points, where available 
3190                      if ix_before > 0: 
3191                          xsol = bisection(property_func, nullc.array[ix_before-1,0], 
3192                                    nullc.array[ix_after,0], xtol=self.pars.xtol) 
3193                  elif abs(xsol - nullc.array[ix_after,0]) < self.pars.xtol: 
3194                      # corner case where zero is right at a sample point 
3195                      # try again with extended sample points, where available 
3196                      if ix_after < len(nullc.array) - 1: 
3197                          xsol = bisection(property_func, nullc.array[ix_before,0], 
3198                                    nullc.array[ix_after+1,0], xtol=self.pars.xtol) 
3199                  x_range[0] = xsol 
3200                  y_range[0] = nullc(xsol) 
3201   
3202          # Right search 
3203          if center_ix == self.pars.all_zone_ixs[-1]: 
3204              # everything to the right is already included 
3205              x_range[1] = nullc.array[-1,0] 
3206              y_range[1] = nullc.array[-1,1] 
3207          else: 
3208              # all sample points with indices center_ix:ix_before (inclusive) 
3209              # have the same property. 
3210              ix_before = self.pars.all_zone_ixs[zone_position+1] 
3211              ix_after = ix_before + 1 
3212              if nullc.array[ix_before,0] > max_zone_x: 
3213                  x_range[1] = max_zone_x 
3214                  y_range[1] = nullc(x_range[1]) 
3215              else: 
3216                  # search between these points on the nullcline 
3217                  xsol = bisection(property_func, nullc.array[ix_before,0], 
3218                                    nullc.array[ix_after,0], xtol=self.pars.xtol) 
3219                  if abs(xsol - nullc.array[ix_before,0]) < self.pars.xtol: 
3220                      # corner case where zero is right at a sample point 
3221                      # try again with extended sample points, where available 
3222                      if ix_before > 0: 
3223                          xsol = bisection(property_func, nullc.array[ix_before-1,0], 
3224                                    nullc.array[ix_after,0], xtol=self.pars.xtol) 
3225                  elif abs(xsol - nullc.array[ix_after,0]) < self.pars.xtol: 
3226                      # corner case where zero is right at a sample point 
3227                      # try again with extended sample points, where available 
3228                      if ix_after < len(nullc.array) - 1: 
3229                          xsol = bisection(property_func, nullc.array[ix_before,0], 
3230                                    nullc.array[ix_after+1,0], xtol=self.pars.xtol) 
3231                  x_range[1] = xsol 
3232                  y_range[1] = nullc(xsol) 
3233   
3234          return [np.array(x_range), np.array(y_range)]

3235   
3236   
3237  # Implementations 


3238 -class fixedpoint_zone(nullcline_zone_leaf):


3239      # needs *two* nullcline objects and a f.p. object 
3240      pass

3241   
3242   
3243  # define prior to node so that can provide leaf class name to node class 


3244 -class inflection_zone_leaf(nullcline_zone_leaf):


3245      """A single inflection point zone. 
3246   
3247      pars: 
3248        xtol --> precision of zone center finding 
3249        find_exact -> exact x value of inflection (not implemented yet) 
3250        zone_width -> positive scalar (default Inf grows to next inflection point) 
3251      """ 
3252      #Only use one of zone_radius or zone_width, otherwise zone_width will be used. 


3253 -    def evaluate(self, nullc):


3254          # Indices of inflection mark last known sample point having 
3255          # same concavity to those locally on its left in the point order 
3256          # from left to right. 
3257          center_ix, x_center, y_center = self._prepare(nullc) 
3258          self.results.x_center = x_center 
3259          self.results.y_center = y_center 
3260          self.results.zone = self._grow_zone_by_width(center_ix, x_center, y_center, nullc, 
3261                                                       nullc.concavity) 
3262          # if got this far, succeeded in finding a zone 
3263          return True

3264   
3265   


3266 -class inflection_zone_node(nullcline_zone_node):


3267      """Find all inflection point zones 
3268      """ 
3269      _leaf_class = inflection_zone_leaf 
3270   


3271 -    def evaluate(self, nullc):


3272          """Assumes at least 2 sample points in nullcline. 
3273          """ 
3274          # Find all approximate places (sample points) where 
3275          # inflections occur. 
3276          # Concavities are -1, 0, 1 but we don't care about the signs 
3277          # so much as their changes. To this end, replace all zeros 
3278          # with their previous neighbor's value 
3279          xarray = nullc.sample_x_domain(refine=self.pars.refine) 
3280          concs = nullc.concavity(xarray) 
3281          conc_zeros = concs == 0 
3282          while np.sometrue(conc_zeros): 
3283              concs_list = list(concs) 
3284              # find lowest index with a zero 
3285              zero_ix = concs_list.index(0) 
3286              if zero_ix > 0: 
3287                  concs[zero_ix] = concs[zero_ix-1] 
3288              else: 
3289                  # replace with next non-zero value 
3290                  try: 
3291                      pos_ix = concs_list.index(1) 
3292                  except ValueError: 
3293                      # no +1's 
3294                      pos_ix = np.inf 
3295                  try: 
3296                      neg_ix = concs_list.index(-1) 
3297                  except ValueError: 
3298                      # no -1's 
3299                      neg_ix = np.inf 
3300                  min_ix = min( pos_ix, neg_ix ) 
3301                  if isfinite(min_ix): 
3302                      concs[0] = concs[min_ix] 
3303                  else: 
3304                      concs[0] = 1 
3305              conc_zeros = concs == 0 
3306          # ignore changes within a single sample point of the endpoints 
3307          # only -1 indicate. The extra -1 subtraction ensures all the +1's 
3308          # become zero so that argwhere can discount those locations. 
3309          zone_center_ixs = np.argwhere(concs[:-1] * concs[1:] - 1).flatten() 
3310          # restore to native x array of nullcline (in case of refinement) 
3311          zone_center_ixs = np.unique(nullc.array[:,0].searchsorted(xarray[zone_center_ixs])) 
3312          self.results.zone_center_ixs = _filter_consecutive(zone_center_ixs, 
3313                                                      minimize_values=concs) 
3314          return len(self.results.zone_center_ixs) > 0

3315   
3316   
3317   


3318 -class max_curvature_zone_leaf(nullcline_zone_leaf):


3319      """A single zone of locally maximal curvature 
3320   
3321      pars: 
3322        xtol --> precision of zone center finding 
3323        direction (currently ignored -- looks for both) 
3324        min_curvature_pc --> percentage of the maximum curvature found at sample 
3325           points for which a local minimum value of curvature must meet to be 
3326           considered a zone. 
3327        find_exact -> exact x value of max curvature (not implemented yet) 
3328        zone_width -> positive scalar (default Inf grows to next local maximum) 
3329      """ 
3330      #Only use one of zone_radius or zone_width, otherwise zone_width will be used. 


3331 -    def evaluate(self, nullc):


3332          # Indices of max curvature mark last known sample point having 
3333          # same curvature gradient to those locally on its left in the point order 
3334          # from left to right. 
3335          center_ix, x_center, y_center = self._prepare(nullc) 
3336          self.results.x_center = x_center 
3337          self.results.y_center = y_center 
3338          self.results.zone = self._set_mutex_zone_by_width(center_ix, x_center, 
3339                                                            y_center, nullc) 
3340          # if got this far, succeeded in finding a zone 
3341          return True

3342   
3343   


3344 -class max_curvature_zone_node(nullcline_zone_node):


3345      """Find all zones with locally maximal curvature. 
3346      Optional selection of those facing up or down only: 
3347      par: direction (-1, 0, 1, where 0 selects either (default)) 
3348      """ 
3349      _leaf_class = max_curvature_zone_leaf 
3350   


3351 -    def evaluate(self, nullc):


3352          try: 
3353              dirn = self.pars.direction 
3354          except AttributeError: 
3355              dirn = 0  # both 
3356          xarray = nullc.sample_x_domain(refine=self.pars.refine) 
3357          gcurvature = nullc.grad_curvature(xarray) 
3358          # find local maxima, including endpoint maxima 
3359          # TEMP: don't look at endpoints for now 
3360          # inside endpoints, look for where d/dx( curvature ) = 0 
3361          # look for sign changes, just like concavity signs 
3362          # for inflection feature 
3363          gsigns = np.sign( gcurvature ) 
3364          gsign_zeros = gsigns == 0 
3365          while np.sometrue(gsign_zeros): 
3366              gsigns_list = list(gsigns) 
3367              # find lowest index with a zero 
3368              zero_ix = gsigns_list.index(0) 
3369              if zero_ix > 0: 
3370                  gsigns[zero_ix] = gsigns[zero_ix-1] 
3371              else: 
3372                  # replace with next non-zero value 
3373                  try: 
3374                      pos_ix = gsigns_list.index(1) 
3375                  except ValueError: 
3376                      # no +1's 
3377                      pos_ix = np.inf 
3378                  try: 
3379                      neg_ix = gsigns_list.index(-1) 
3380                  except ValueError: 
3381                      # no -1's 
3382                      neg_ix = np.inf 
3383                  min_ix = min( pos_ix, neg_ix ) 
3384                  if isfinite(min_ix): 
3385                      gsigns[0] = gsigns[min_ix] 
3386                  else: 
3387                      gsigns[0] = 1 
3388              gsign_zeros = gsigns == 0 
3389          # ignore changes within a single sample point of the endpoints 
3390          # only -1 indicate. The extra -1 subtraction ensures all the +1's 
3391          # become zero so that argwhere can discount those locations. 
3392          candidate_ixs = np.argwhere(gsigns[:-1] * gsigns[1:] - 1).flatten() 
3393          curvature = nullc.curvature(xarray) 
3394          if dirn == 0: 
3395              max_curvature = max(abs(curvature)) 
3396              def test(c, thresh): 
3397                  return abs(c) > thresh

3398          elif dirn == -1: 
3399              # concave down only 
3400              max_curvature = min([0, min(curvature)]) 
3401              def test(c, thresh): 
3402                  return c < thresh

3403          elif dirn == 1: 
3404              # concave up only 
3405              max_curvature = max([0, max(curvature)]) 
3406              def test(c, thresh): 
3407                  return c > thresh 
3408          else: 
3409              raise ValueError("Invalid direction given") 
3410          try: 
3411              min_curvature_pc = self.pars.min_curvature_pc 
3412          except AttributeError: 
3413              min_curvature_pc = 0 
3414          curvature_thresh = min_curvature_pc*max_curvature 
3415          zone_center_ixs = [ix for ix in candidate_ixs if \ 
3416                                     test(curvature[ix], curvature_thresh)] 
3417          # restore to native x array of nullcline (in case of refinement) 
3418          zone_center_ixs = np.unique(nullc.array[:,0].searchsorted(xarray[zone_center_ixs])) 
3419          self.results.zone_center_ixs = _filter_consecutive(zone_center_ixs, 
3420                                                      minimize_values=curvature) 
3421          return len(self.results.zone_center_ixs) > 0 
3422   
3423   
3424  # NOT YET DEFINED 


3425 -class min_curvature_zone(nullcline_zone_leaf):


3426      pass

3427   
3428   
3429   
3430  # --------------------------------------------------------------- 
3431  # PLOTTING and ANIMATION TOOLS 
3432   


3433 -def plot_PP_fps(fps, coords=None, do_evecs=False, markersize=10):


3434      """Draw 2D list of fixed points (singletons allowed), must be 
3435      fixedpoint_2D objects. 
3436   
3437      Optional do_evecs (default False) draws eigenvectors around each f.p. 
3438   
3439      Requires matplotlib 
3440      """ 
3441      if isinstance(fps, fixedpoint_2D): 
3442          # singleton passed 
3443          fps = [fps] 
3444   
3445      x, y = fps[0].fp_coords 
3446      for fp in fps: 
3447          # When matplotlib implements half-full circle markers 
3448          #if fp.classification == 'saddle': 
3449              # half-open circle 
3450          if fp.stability == 'u': 
3451              style = 'wo' 
3452          elif fp.stability == 'c': 
3453              style = 'co' 
3454          else: # 's' 
3455              style = 'ko' 
3456          plt.plot(fp.point[x], fp.point[y], style, markersize=markersize, mew=2)

3457   


3458 -def plot_PP_vf(gen, xname, yname, N=20, subdomain=None, scale_exp=0):


3459      """Draw 2D vector field in (xname, yname) coordinates of given Generator, 
3460      sampling on a uniform grid of n by n points. 
3461   
3462      Optional subdomain dictionary specifies axes limits in each variable, 
3463      otherwise Generator's xdomain attribute will be used. 
3464   
3465      For systems of dimension > 2, the non-phase plane variables will be held 
3466        constant at their initial condition values set in the Generator. 
3467   
3468      Optional scale_exp is an exponent (domain is all reals) which rescales 
3469        size of arrows in case of disparate scales in the vector field. Larger 
3470        values of scale magnify the arrow sizes. For stiff vector fields, values 
3471        from -3 to 3 may be necessary to resolve arrows in certain regions. 
3472   
3473      Requires matplotlib 0.99 or later 
3474      """ 
3475      assert N > 1 
3476      xdom = gen.xdomain[xname] 
3477      ydom = gen.xdomain[yname] 
3478      if subdomain is not None: 
3479          try: 
3480              xdom = subdomain[xname] 
3481          except KeyError: 
3482              pass 
3483          try: 
3484              ydom = subdomain[yname] 
3485          except KeyError: 
3486              pass 
3487      assert all(isfinite(xdom)), "Must specify a finite domain for x direction" 
3488      assert all(isfinite(ydom)), "Must specify a finite domain for y direction" 
3489      w = xdom[1]-xdom[0] 
3490      h = ydom[1]-ydom[0] 
3491   
3492      xdict = gen.initialconditions.copy() 
3493   
3494      xix = gen.funcspec.vars.index(xname) 
3495      yix = gen.funcspec.vars.index(yname) 
3496   
3497      xs = np.linspace(xdom[0], xdom[1], N) 
3498      ys = np.linspace(ydom[0], ydom[1], N) 
3499   
3500      X, Y = np.meshgrid(xs, ys) 
3501      dxs, dys = np.meshgrid(xs, ys) 
3502   
3503  ##    dx_big = 0 
3504  ##    dy_big = 0 
3505      dz_big = 0 
3506      vec_dict = {} 
3507   
3508  #    dxs = array((n,), float) 
3509  #    dys = array((n,), float) 
3510      for xi, x in enumerate(xs): 
3511          for yi, y in enumerate(ys): 
3512              xdict.update({xname: x, yname: y}) 
3513              dx, dy = gen.Rhs(0, xdict)[[xix, yix]] 
3514              # note order of indices 
3515              dxs[yi,xi] = dx 
3516              dys[yi,xi] = dy 
3517              dz = np.linalg.norm((dx,dy)) 
3518  ##            vec_dict[ (x,y) ] = (dx, dy, dz) 
3519  ##            if dx > dx_big: 
3520  ##                dx_big = dx 
3521  ##            if dy > dy_big: 
3522  ##                dy_big = dy 
3523              if dz > dz_big: 
3524                  dz_big = dz 
3525   
3526      plt.quiver(X, Y, dxs, dys, angles='xy', pivot='middle', units='inches', 
3527                 scale=dz_big*max(h,w)/(10*exp(2*scale_exp)), lw=0.01/exp(scale_exp-1), 
3528                 headwidth=max(2,1.5/(exp(scale_exp-1))), 
3529                 #headlength=2*max(2,1.5/(exp(scale_exp-1))), 
3530                 width=0.001*max(h,w), minshaft=2, minlength=0.001) 
3531   
3532  ##    # Use 95% of interval size 
3533  ##    longest_x = w*0.95/(n-1) 
3534  ##    longest_y = h*0.95/(n-1) 
3535  ##    longest = min(longest_x, longest_y) 
3536  ## 
3537  ##    scaling_x = longest_x/dx_big 
3538  ##    scaling_y = longest_y/dy_big 
3539  ##    scaling = min(scaling_x, scaling_y) 
3540   
3541      ax = plt.gca() 
3542  ##    hw = longest/10 
3543  ##    hl = hw*2 
3544  ##    for x in xs: 
3545  ##        for y in ys: 
3546  ##            dx, dy, dz = vec_dict[ (x,y) ] 
3547  ##            plt.arrow(x, y, scaling*dx, yscale*scaling*dy, 
3548  ##                      head_length=hl, head_width=hw, length_includes_head=True) 
3549      ax.set_xlim(xdom) 
3550      ax.set_ylim(ydom) 
3551      plt.draw()

3552   
3553   


3554 -def get_PP(gen, pt, vars, doms=None, doplot=True, 
3555             t=0, saveplot=None, format='svg', trail_pts=None, 
3556             null_style='-', orbit_col='g', orbit_style='-'):


3557      """Get a phase plane for generator gen at point pt. 
3558      Specify t if the system is non-autonomous. 
3559      If trail_pts are given (e.g., via show_PPs) then these are added 
3560      behind the point pt. 
3561   
3562      Requires matplotlib 
3563      """ 
3564      xFS, yFS = gen._FScompatibleNames(vars) 
3565      x, y = gen._FScompatibleNamesInv(vars) 
3566      pt = gen._FScompatibleNamesInv(pt) 
3567      ptFS = gen._FScompatibleNames(pt) 
3568   
3569      sub_dom = filteredDict(dict(ptFS), 
3570                             remain(gen.funcspec.vars, [x,y])) 
3571      if doms is None: 
3572          doms = gen.xdomain 
3573      else: 
3574          doms = gen._FScompatibleNames(doms) 
3575      sub_dom[xFS] = doms[xFS] 
3576      sub_dom[yFS] = doms[yFS] 
3577      x_dom = doms[xFS] 
3578      y_dom = doms[yFS] 
3579      x_interval = Interval(xFS, float, x_dom, abseps=0) 
3580      y_interval = Interval(yFS, float, y_dom, abseps=0) 
3581      fps = find_fixedpoints(gen, n=6, subdomain=sub_dom, 
3582                             t=t, eps=1e-8) 
3583   
3584      f = figure(1) 
3585      nulls_x, nulls_y, handles = find_nullclines(gen, xFS, yFS, 
3586                                      x_dom=x_dom, y_dom=y_dom, 
3587                                      fixed_vars=ptFS, n=3, t=t, 
3588                                      max_step={xFS: 0.1, yFS: 1}, 
3589                                      max_num_points=10000, fps=fps, 
3590                                      doplot=doplot, plot_style=null_style, 
3591                                      newfig=False) 
3592      if doplot: 
3593          tol = 0.01 
3594          xwidth = abs(x_dom[1]-x_dom[0]) 
3595          ywidth = abs(y_dom[1]-y_dom[0]) 
3596          x_lims = [x_dom[0]-tol*xwidth, x_dom[1]+tol*xwidth] 
3597          y_lims = [y_dom[0]-tol*ywidth, y_dom[1]+tol*ywidth] 
3598          if trail_pts is not None: 
3599              pp.plot(trail_pts[x], trail_pts[y], orbit_col) 
3600          pp.plot(pt[x], pt[y], orbit_col+'o') 
3601          for fp in fps: 
3602              if fp[x] in x_interval and fp[y] in y_interval: 
3603                  pp.plot(fp[x], fp[y], 'ko') 
3604          ## the following commands can introduce artifact lines!! 
3605          ax = pp.gca() 
3606          ax.set_xlim(x_lims) 
3607          ax.set_ylim(y_lims) 
3608          draw() 
3609          ## 
3610          if saveplot is not None: 
3611              if saveplot[-4:] != '.' + format: 
3612                  saveplot = saveplot + '.' + format 
3613              f.savefig(saveplot, format=format) 
3614              f.clf() 
3615      return fps, nulls_x, nulls_y

3616   
3617   


3618 -def show_PPs(gen, x, y, traj, t_start, t_step, t_end, moviename=None, doms=None, 
3619               show_trail=False, add_traj=None):


3620      """Show phase plane x vs y for Generator gen over trajectory traj, 
3621      over times t_start:t_step:t_end. 
3622      Option to create a movie, provided ffmpeg if installed. 
3623      Optional domain dictionary for variables can be provided, otherwise their 
3624      extents in the given trajectory will be used. 
3625      Option to show trail behind green orbit (default False). 
3626      Option to add a comparison trajectory / nullclines to the plot. 
3627   
3628      Requires matplotlib and ffmpeg 
3629      """ 
3630      t = t_start 
3631      pt0 = traj(t_start) 
3632      pt1 = traj(t_end) 
3633      xs = [pt0[x], pt1[x]] 
3634      ys = [pt0[y], pt1[y]] 
3635      if doms is None: 
3636          doms = {x: [min(xs),max(xs)], y: [min(ys),max(ys)]} 
3637      if moviename is not None: 
3638          os.system('mkdir %s' % moviename) 
3639          os.chdir(moviename) 
3640      i = 0 
3641      while t <= t_end: 
3642          pt = traj(t) 
3643          i += 1 
3644          print "Frame %i, t = %.4f: step %.4f until %.4f" % (i, t, t_step, t_end) 
3645          if moviename is not None: 
3646              saveplot = 'pp_fig_%03d' % i 
3647              format = 'png' 
3648          else: 
3649              saveplot = None 
3650              format = 'svg' 
3651          if show_trail and t > t_start: 
3652              trail_pts = traj.sample(tlo=t_start, thi=t) 
3653              if add_traj is not None: 
3654                  trail_pts2 = add_traj.sample(tlo=t_start, thi=t) 
3655          else: 
3656              trail_pts = None 
3657              trail_pts2 = None 
3658          if add_traj is None: 
3659              get_PP(gen, pt, [x, y], doms=doms, 
3660                 t=t, doplot=True, saveplot=saveplot, format=format, 
3661                 trail_pts=trail_pts) 
3662          else: 
3663              get_PP(gen, pt, [x, y], doms=doms, 
3664                 t=t, doplot=True, saveplot=None, 
3665                 trail_pts=trail_pts, null_style='-', orbit_col='g', orbit_style='-') 
3666              get_PP(gen, add_traj(t), [x, y], doms=doms, 
3667                 t=t, doplot=True, saveplot=saveplot, format=format, 
3668                 trail_pts=trail_pts2, null_style=':', orbit_col='c', orbit_style='-') 
3669          t += t_step 
3670      if moviename is not None: 
3671          # copy the final frame a few times to pad out end of video 
3672          for j in range(i+1, i+51): 
3673              os.system('cp pp_fig_%03d.png pp_fig_%03d.png' % (i, j)) 
3674          os.system('ffmpeg -f image2 -i pp_fig_%03d.png -r 14 pp_movie.avi') 
3675          os.chdir('..')

3676   
3677   
3678  # ----------------------------------------------------------- 
3679   
3680  # EXPERIMENTAL CLASS 


3681 -class mesh_patch_2D(object):


3682      """2D mesh patch generator (for 4 or 8 points of a fixed distance 
3683      from a central point). i.e., total number of mesh points may be 5 
3684      or 9. 
3685   
3686      Once either 5 or 9 points have been selected, this is fixed for 
3687      the mesh patch instance. The central point p0 and radii r of the 
3688      mesh points are mutable using the 'update' method. If 
3689      shape='circle' (default), mesh points are equidistant from p0. If 
3690      shape='square', mesh points are distributed around the corners and 
3691      mid-sides of a square of side length 2*r. 
3692   
3693      The points are in the order p0 followed by points in 'clockwise' 
3694      order in the phase plane defined by the two variable names in p0. 
3695   
3696      Functions / callable objects can be applied to all points in the 
3697      mesh patch by calling the mesh patch's 'eval' method with the 
3698      function. It returns an a dictionary of mesh points -> function 
3699      return values. 
3700   
3701      The direction of minimum (interpolated) gradient of a functional 
3702      over the mesh can be determined from the 'min_gradient_dir' method. 
3703      A similar method 'max_gradient_dir' also exists. 
3704   
3705      Highly experimental class! 
3706   
3707      Rob Clewley, Jan 2007 
3708      """ 
3709      _s2 = sqrt(2)/2 
3710      _mesh5 = [(0,0), (0,1), (1,0), (0,-1), (-1,0)] 
3711      _mesh9s = [(0,0), (0,1), (1,1), (1,0), (1,-1), 
3712                 (0,-1), (-1,-1), (-1,0), (-1, 1)] 
3713      _mesh9c = [(0,0), (0,1), (_s2,_s2), (1,0), (_s2,-_s2), 
3714                 (0,-1), (-_s2,-_s2), (-1,0), (-_s2,_s2)] 
3715   


3716 -    def __init__(self, p0, r, n=5, shape='circle'):


3717          """p0 should be of type Point, r must be either a scalar or a Point 
3718          giving weighted radius in each of two directions, n must be 5 or 9. 
3719          """ 
3720          self.p0 = p0 
3721          try: 
3722              if len(r) == 2: 
3723                  assert r.coordnames == p0.coordnames, "Coordinate names of "\ 
3724                         "r and p0 must match" 
3725          except TypeError: 
3726              # r is a scalar 
3727              pass 
3728          self.r = r 
3729          self.n = n 
3730          self.shape = shape 
3731          self.make_mesh(makelocalmesh=True) 
3732          self.derive_angles()

3733   


3734 -    def make_mesh(self, makelocalmesh=False):


3735          if self.n==5: 
3736              mesh = [self.r*array(p, 'd') for p in self._mesh5] 
3737              self.shape = 'circle'    # force it regardless of its value 
3738          elif self.n==9: 
3739              if self.shape=='circle': 
3740                  m = self._mesh9c 
3741              elif self.shape=='square': 
3742                  m = self._mesh9s 
3743              else: 
3744                  raise ValueError("Shape argument must be 'square' or 'circle'") 
3745              mesh = [self.r*array(p, 'd') for p in m] 
3746          else: 
3747              raise ValueError("Only n=5 or 9 meshes are supported") 
3748          # self.mesh is the absolute coords of the mesh 
3749          try: 
3750              self.mesh = pointsToPointset([self.p0+p for p in mesh]) 
3751          except AssertionError: 
3752              raise TypeError("Central point must be given by a 2D Point object") 
3753          if makelocalmesh: 
3754              # Only needs to be done once 
3755              # Make (0,0) as a Point with the correct coordnames, for convenience 
3756              zp = self.p0*0 
3757              # self.mesh_local is the mesh in local coordinates (relative to p0) 
3758              self.mesh_local = pointsToPointset([zp+p for p in mesh])

3759   


3760 -    def update(self, p0=None, r=None):


3761          """Change the central point p0 or patch radius r 
3762          """ 
3763          do_update = False 
3764          if p0 is not None: 
3765              self.p0 = p0 
3766              do_update = True 
3767          if r is not None: 
3768              self.r = r 
3769              do_update = True 
3770          if do_update: 
3771              self.make_mesh()

3772   


3773 -    def derive_angles(self):


3774          # angles are always measured in the positive direction here 
3775          # angles don't include the centre point at (0,0), and are measured 
3776          # from the "x" axis 
3777          if self.n == 5: 
3778              self._angles = [pi/2, 0, 3*pi/2, pi] 
3779          else: 
3780              # only mesh-points not aligned with axes need to be calculated 
3781              # the others are constant regardless of re-scaling of axes 
3782              vars = self.p0.coordnames 
3783              self._angles = [] 
3784              for p in self.mesh_local[1:]: 
3785                  a = atan2(p[vars[1]],p[vars[0]]) 
3786                  if a < 0: 
3787                      a += 2*pi 
3788                  self._angles.append(a)

3789   
3790   


3791 -    def __getitem__(self, ix):


3792          return self.mesh[ix]

3793   


3794 -    def eval(self, f):


3795          """Evaluate the function or callable object f at the mesh patch points. 
3796          Returns a dictionary of 
3797              mesh point index -> function value 
3798          Indices are always in the same order as the Pointset attribute 'mesh' 
3799          """ 
3800          res = {} 
3801          for i, p in enumerate(self.mesh): 
3802              try: 
3803                  res[i] = f(p) 
3804              except: 
3805                  print "Problem evaluating supplied function on mesh patch at " \ 
3806                      "point ", p 
3807                  raise 
3808          return res

3809   


3810 -    def setup_grad(self, valdict, orient, dir):


3811          try: 
3812              vals = [valdict[i] for i in range(self.n)] 
3813          except KeyError: 
3814              raise ValueError("vals must have same length as number " 
3815                               "of mesh points") 
3816          if orient is not None and dir is not None: 
3817              ixs = [] 
3818              try: 
3819                  vars = orient.coordnames 
3820              except AttributeError: 
3821                  raise TypeError("orient must be a vector (Point object)") 
3822              if dir not in [-1,1]: 
3823                  raise ValueError("dir value must be 1 or -1") 
3824              x = dir*orient[vars[0]] 
3825              y = dir*orient[vars[1]] 
3826              # angle of directed orientation 
3827              theta = atan2(y,x) 
3828              if theta < 0: 
3829                  theta += 2*pi 
3830              # filter out mesh points whose defining angles are outside of 
3831              # the half-plane specified by the normal vector 
3832              twopi = 2*pi 
3833              for i, a in enumerate(self._angles): 
3834                  test = abs(a - theta) 
3835                  if test <= pi/2 or twopi-test <= pi/2: 
3836                      # append 1+i to correspond to 1..n mesh points 
3837                      ixs.append(i+1) 
3838  ##            print "Theta was ", theta 
3839  ##            print "Angles: ", self._angles 
3840  ##            print "Min grad using ixs", ixs 
3841              use_vals = [vals[i] for i in ixs] 
3842          else: 
3843              ixs = range(1,self.n) 
3844              use_vals = vals 
3845          return vals, ixs, use_vals

3846   


3847 -    def min_gradient_dir(self, valdict, orient=None, dir=None):


3848          """Find the direction of any local minima of a functional over 
3849          the mesh patch, whose dictionary of 
3850              mesh point index -> function value 
3851          is given by valdict. The magnitudes of these values are 
3852          compared. The direction is indicated by a Point object, a 
3853          relative position at the patch's characteristic radius from 
3854          p0. 
3855   
3856          If the functional is constant everywhere on the patch (degenerate) 
3857          then a random direction is returned. If the functional is constant 
3858          everywhere except at the centre, then the vector (0,0) is returned 
3859          if the centre has lower value; a random vector is returned 
3860          otherwise. 
3861   
3862          If the functional is partially degenerate on some subset of 
3863          mesh points then the optional orient and dir arguments can 
3864          provide a means to select, provided the mesh resolution is 
3865          sufficiently high. 
3866   
3867          orient is a vector (Point object) relative to p0 orienting a 
3868          "positive" direction, such that the integer dir = -1 or +1 
3869          selects one half of the mesh points on which to conduct the 
3870          search. 
3871          """ 
3872          return self.__min_grad(valdict, orient, dir, 1)

3873   


3874 -    def min_gradient_mesh(self, valdict, orient=None, dir=None):


3875          """Find the direction of any local minima of a functional over 
3876          the mesh patch, whose dictionary of 
3877              mesh point index -> function value 
3878          is given by valdict. The magnitudes of these values are 
3879          compared. The direction is returned as a pair containing the two 
3880          closest mesh points to the direction (in mesh local coords). 
3881   
3882          If the functional is constant everywhere on the patch (degenerate) 
3883          then a random direction is returned. If the functional is constant 
3884          everywhere except at the centre, then the vector (0,0) is returned 
3885          if the centre has lower value; a random vector is returned 
3886          otherwise. 
3887   
3888          If the functional is partially degenerate on some subset of 
3889          mesh points then the optional orient and dir arguments can 
3890          provide a means to select, provided the mesh resolution is 
3891          sufficiently high. 
3892   
3893          orient is a vector (Point object) relative to p0 orienting a 
3894          "positive" direction, such that the integer dir = -1 or +1 
3895          selects one half of the mesh points on which to conduct the 
3896          search. 
3897          """ 
3898          return self.__min_grad(valdict, orient, dir, 0)

3899   


3900 -    def __min_grad(self, valdict, orient, dir, return_type):


3901          vals, ixs, use_vals = self.setup_grad(valdict, orient, dir) 
3902          degen_everywhere = alltrue([v==vals[0] for v in vals]) 
3903          degen_exterior = alltrue([v==vals[ixs[0]] for v in use_vals]) 
3904          degen_exterior_lower = degen_exterior and vals[0] >= vals[ixs[0]] 
3905          degen_exterior_higher = degen_exterior and vals[0] < vals[ixs[0]] 
3906          if degen_everywhere or degen_exterior_lower: 
3907              # degenerate case -- return random vector 
3908              x = uniform(-1,1) 
3909              y = uniform(-1,1) 
3910              vl = sqrt(x*x + y*y) 
3911              x = x/vl 
3912              y = y/vl 
3913              if return_type == 0: 
3914                  raise RuntimeError("No valid directions to point in") 
3915              else: 
3916                  return Point(dict(zip(self.p0.coordnames, [x,y]))) 
3917          elif degen_exterior_higher: 
3918              if return_type == 0: 
3919                  raise RuntimeError("No valid directions to point in") 
3920              else: 
3921                  return Point(dict(zip(self.p0.coordnames, [0,0]))) 
3922          d1 = (Inf, NaN) 
3923          d2 = (Inf, NaN) 
3924          for i, v in enumerate(use_vals): 
3925              if ixs[i] == 0: 
3926                  # don't include centre point 
3927                  continue 
3928              if abs(v) < d1[0]: 
3929                  d2 = d1 
3930                  d1 = (abs(v), ixs[i]) 
3931              elif abs(v) < d2[0]: 
3932                  d2 = (abs(v), ixs[i]) 
3933          # the two best mesh patch points (unless partially degenerate) 
3934          pt1 = self.mesh_local[d1[1]] 
3935          pt2 = self.mesh_local[d2[1]] 
3936          if return_type == 0: 
3937              return (pt1, pt2) 
3938          else: 
3939              score1 = d1[0] 
3940              score2 = d2[0] 
3941              if score1 + score2 == 0: 
3942                  interp_pt = pt1+pt2 
3943                  return self.r*interp_pt/norm(interp_pt) 
3944              elif score1 == 0: 
3945                  return pt1 
3946              elif score2 == 0: 
3947                  return pt2 
3948              else: 
3949                  interp_pt = pt1/score1+pt2/score2 
3950                  return self.r*interp_pt/norm(interp_pt)

3951   
3952   


3953 -    def max_gradient_dir(self, valdict, orient=None, dir=None):


3954          """Find the direction of maximum gradient of a functional over 
3955          the mesh patch, whose dictionary of 
3956              mesh point index -> function value 
3957          is given by valdict. The magnitudes of the values are 
3958          compared. The direction is indicated by a Point object, a 
3959          relative position at the patch's characteristic radius from 
3960          p0. 
3961   
3962          If the functional is constant everywhere on the patch (degenerate) 
3963          then a random direction is returned. If the functional is constant 
3964          everywhere except at the centre, then the vector (0,0) is returned 
3965          if the centre has higher value; a random vector is returned 
3966          otherwise. 
3967   
3968          If the functional is partially degenerate on some subset of 
3969          mesh points then the optional orient and dir arguments can 
3970          provide a means to select, provided the mesh resolution is 
3971          sufficiently high. 
3972   
3973          orient is a vector (Point object) relative to p0 orienting a 
3974          "positive" direction, such that the integer dir = -1 or +1 
3975          selects one half of the mesh points on which to conduct the 
3976          search. 
3977          """ 
3978          return self.__max_grad(valdict, orient, dir, 1)

3979   


3980 -    def max_gradient_mesh(self, valdict, orient=None, dir=None):


3981          """Find the direction of maximum gradient of a functional over 
3982          the mesh patch, whose dictionary of 
3983              mesh point index -> function value 
3984          is given by valdict. The magnitudes of the values are 
3985          compared. The direction is returned as a pair containing the two 
3986          closest mesh points to the direction (in mesh local coords). 
3987   
3988          If the functional is constant everywhere on the patch (degenerate) 
3989          then a random direction is returned. If the functional is constant 
3990          everywhere except at the centre, then the vector (0,0) is returned 
3991          if the centre has higher value; a random vector is returned 
3992          otherwise. 
3993   
3994          If the functional is partially degenerate on some subset of 
3995          mesh points then the optional orient and dir arguments can 
3996          provide a means to select, provided the mesh resolution is 
3997          sufficiently high. 
3998   
3999          orient is a vector (Point object) relative to p0 orienting a 
4000          "positive" direction, such that the integer dir = -1 or +1 
4001          selects one half of the mesh points on which to conduct the 
4002          search. 
4003          """ 
4004          return self.__max_grad(valdict, orient, dir, 0)

4005   


4006 -    def __max_grad(self, valdict, orient, dir, return_type):


4007          vals, ixs, use_vals = self.setup_grad(valdict, orient, dir) 
4008          degen_everywhere = alltrue([v==vals[0] for v in vals]) 
4009          degen_exterior = alltrue([v==vals[1] for v in use_vals]) 
4010          degen_exterior_lower = degen_exterior and vals[0] >= vals[ixs[0]] 
4011          degen_exterior_higher = degen_exterior and vals[0] < vals[ixs[0]] 
4012          if degen_everywhere or degen_exterior_higher: 
4013              # degenerate case -- return random vector 
4014              x = uniform(-1,1) 
4015              y = uniform(-1,1) 
4016              vl = sqrt(x*x + y*y) 
4017              x = x/vl 
4018              y = y/vl 
4019              if return_type == 0: 
4020                  raise RuntimeError("No valid directions to point in") 
4021              else: 
4022                  return Point(dict(zip(self.p0.coordnames, [x,y]))) 
4023          elif degen_exterior_lower: 
4024              if return_type == 0: 
4025                  raise RuntimeError("No valid directions to point in") 
4026              else: 
4027                  return Point(dict(zip(self.p0.coordnames, [0,0]))) 
4028          d1 = (0, NaN) 
4029          d2 = (0, NaN) 
4030          for i, v in enumerate(use_vals): 
4031              if ixs[i] == 0: 
4032                  # don't include centre point 
4033                  continue 
4034              if abs(v) > d1[0]: 
4035                  d2 = d1 
4036                  d1 = (abs(v), ixs[i]) 
4037              elif abs(v) > d2[0]: 
4038                  d2 = (abs(v), ixs[i]) 
4039          # the two best mesh patch points (unless partially degenerate) 
4040          pt1 = self.mesh_local[d1[1]] 
4041          pt2 = self.mesh_local[d2[1]] 
4042          if return_type == 0: 
4043              return (pt1, pt2) 
4044          else: 
4045              score1 = d1[0] 
4046              score2 = d2[0] 
4047              interp_pt = pt1*score1+pt2*score2 
4048              return self.r*interp_pt/norm(interp_pt)

4049   


4050 -    def __repr__(self):


4051          return "Mesh patch [" + ", ".join(["(" + \ 
4052                                  str(m).replace(' ','').replace('\n',', ')+")" \ 
4053                                  for m in self.mesh]) +"]"

4054   
4055      __str__ = __repr__

4056   
4057   
4058   
4059  # ----------------------------------------------------------------------------- 
4060   
4061  ## PRIVATE CLASSES, FUNCTIONS, and CONSTANTS 
4062   
4063  # For use when operations don't allow symbol Inf 
4064  _num_inf = 1.0e100 
4065   
4066   


4067 -def create_test_fn(gen, tmax, dist_pts):


4068      """Create integration test function using the supplied generator. 
4069   
4070      The test function will return a dictionary of the two closest 
4071      points (keys 1 and 2) mapping to their respective distances and 
4072      pointset index positions. 
4073      """ 
4074   
4075      gen.set(tdata=[0,tmax]) 
4076   
4077      def Tn_integ(ic): 
4078          gen.set(ics=ic) 
4079          try: 
4080              test_traj = gen.compute('test') 
4081          except: 
4082              print "Problem integrating test trajectory at i.c. ", ic 
4083              raise 
4084          test_pts = test_traj.sample(coords=dist_pts.all_pts.coordnames) 
4085          # distance of endpoint to pointset 
4086          try: 
4087              d_info = dist_pts(test_pts[-1], use_norm=True, minmax=['min']) 
4088          except ValueError: 
4089              # This error happens when fsolve tries initial conditions that 
4090              # break the integrator 
4091              return (_num_inf,NaN) 
4092          pos = d_info['min'][1]['pos'] 
4093          return (test_pts[-1]-dist_pts.all_pts[pos], pos)

4094      return Tn_integ 
4095   
4096   


4097 -def create_test_fn_with_events(gen, tmax, dist_pts, iso_ev, other_evnames, pars_to_vars):


4098      """Create integration test function using the supplied generator, 
4099      assuming it contains isochron-related events. 
4100   
4101      The test function will return a dictionary of the two closest 
4102      points (keys 1 and 2) mapping to their respective distances and 
4103      pointset index positions. 
4104      """ 
4105   
4106      def Tn_integ(ic): 
4107          gen.set(ics=ic, tdata=[0,tmax]) 
4108          try: 
4109              test_traj = gen.compute('test') 
4110          except: 
4111              print "Problem integrating test trajectory at i.c. ", ic 
4112              raise 
4113          test_pts = test_traj.sample(coords=dist_pts.all_pts.coordnames) 
4114          # distance of endpoint to pointset 
4115          try: 
4116              d_info = dist_pts(test_pts[-1], use_norm=True, minmax=['min']) 
4117          except ValueError: 
4118              # This error happens when fsolve tries initial conditions that 
4119              # break the integrator 
4120              return (_num_inf,NaN) 
4121          # refine min position using isochron-related events 
4122          q=test_pts[-1] 
4123          perp_ev, t_ev = _find_min_pt(gen, q, 
4124                                       d_info['min'][1]['pos'], dist_pts.all_pts, 
4125                                       pars_to_vars, iso_ev, other_evnames) 
4126          ev_pt = perp_ev[0][q.coordnames] 
4127          # different return format to version w/o events 
4128          return (test_pts[-1]-ev_pt, t_ev, ev_pt)

4129      return Tn_integ 
4130   
4131   
4132   


4133 -def _xinf_ND(xdot,x0,args=(),xddot=None,xtol=1.49012e-8):


4134      """Private function for wrapping the fsolving for x_infinity 
4135      for a variable x in N dimensions""" 
4136      try: 
4137          result = fsolve(xdot,x0,args,fprime=xddot,xtol=xtol,full_output=1) 
4138      except (ValueError, TypeError, OverflowError): 
4139          xinf_val = NaN 
4140      except: 
4141          print "Error in fsolve:", sys.exc_info()[0], sys.exc_info()[1] 
4142          xinf_val = NaN 
4143      else: 
4144          if result[2] in (1,2,3): #,4,5): 
4145              # 4,5 means "not making good progress" (see fsolve docstring) 
4146              xinf_val = float(result[0]) 
4147          else: 
4148              xinf_val = NaN 
4149      return xinf_val

4150   
4151   


4152 -def _xinf_1D(xdot,x0,args=(),xddot=None,xtol=1.49012e-8):


4153      """Private function for wrapping the solving for x_infinity 
4154      for a variable x in 1 dimension""" 
4155      try: 
4156          if xddot is None: 
4157              xinf_val = float(fsolve(xdot,x0,args,xtol=xtol)) 
4158          else: 
4159              xinf_val = float(newton_meth(xdot,x0,fprime=xddot,args=args)) 
4160      except RuntimeError: 
4161          xinf_val = NaN 
4162      return xinf_val

4163   
4164   


4165 -def _find_min_pt(gen, q, pos1, pts, pars_to_vars, iso_ev, other_evnames, 
4166                   offset=4):


4167      ts = pts['t'] 
4168      if pos1 > offset: 
4169          t_pos1n = ts[pos1-offset] 
4170          p = pts[pos1-offset] 
4171      else: 
4172          dt = ts[pos1+1] - ts[pos1] 
4173          t_pos1n = ts[pos1] - offset*dt 
4174          p = pts[pos1-offset]  # OK b/c assume periodic orbit 
4175      if pos1 < len(pts)-offset: 
4176          t_pos1p = ts[pos1+offset] 
4177      else: 
4178          dt = ts[pos1] - ts[pos1-1] 
4179          t_pos1p = ts[pos1] + offset*dt 
4180      tdata_local = [0, t_pos1p-t_pos1n + 1e-5] 
4181      gen.eventstruct.setActiveFlag(iso_ev, True) 
4182      for evn in other_evnames: 
4183          gen.eventstruct.setActiveFlag(evn, False) 
4184      qpars = {} 
4185      for parname, varname in pars_to_vars.items(): 
4186          qpars[parname] = q[varname] 
4187      gen.set(ics=p, tdata=tdata_local, pars=qpars) 
4188  ##    # temp 
4189  ##    vs=dict(p) 
4190  ##    vs['t']=tdata_local[0] 
4191  ##    print "\n" 
4192  ##    print "dist 1 = ", norm(p-q) 
4193  ##    print "ev fn 1 = ", gen.eventstruct.events[iso_ev]._fn(vs, gen.pars) 
4194  ##    print "ptset min dist = ", norm(pts[pos1]-q) 
4195  ##    vs=dict(pts[pos1+2]) 
4196  ##    vs['t']=tdata_local[1] 
4197  ##    print "dist 2 = ", norm(pts[pos1+2]-q) 
4198  ##    print "ev fn 2 = ", gen.eventstruct.events[iso_ev]._fn(vs, gen.pars) 
4199  ##    # end temp 
4200      test_traj2 = gen.compute('test2') 
4201      gen.eventstruct.setActiveFlag(iso_ev, False) 
4202      # assert presence of terminal event 
4203      perp_ev = gen.getEvents()[iso_ev] 
4204      try: 
4205          assert len(perp_ev) == 1 
4206          t_ev = perp_ev['t'][0] + tdata_local[0] 
4207      except: 
4208          print "Event name:", iso_ev 
4209          print perp_ev 
4210          raise ValueError("No nearest point found with event") 
4211      return (perp_ev, t_ev)

4212   
4213   


4214 -class base_n_counter(object):


4215      """Simple counter in base-n, using d digits. 
4216      Resets to zeros when state (n-1, n-1, ..., n-1) is 
4217      incremented. 
4218   
4219      base_n_counter(n, d) 
4220   
4221      Public attribute: 
4222          counter 
4223   
4224      Implements methods: 
4225          inc            -- increment counter (returns nothing) 
4226          __getitem__    -- return ith component of counter 
4227          reset          -- set counter to (0, 0, ..., 0) 
4228      """ 


4229 -    def __init__(self, n, d):


4230          self._maxval = n-1 
4231          self._d = d 
4232          self.counter = np.zeros((d,))

4233   


4234 -    def inc(self):


4235          ix = 0 
4236          while True: 
4237              if ix == self._d: 
4238                  self.counter = np.zeros((self._d,)) 
4239                  break 
4240              if self.counter[ix] < self._maxval: 
4241                  self.counter[ix] += 1 
4242                  break 
4243              else: 
4244                  self.counter[ix] = 0 
4245                  ix += 1

4246   


4247 -    def __getitem__(self, i):


4248          try: 
4249              return self.counter[i] 
4250          except IndexError: 
4251              raise IndexError("Invalid index for counter")

4252   


4253 -    def reset(self):


4254          self.counter = np.zeros((self._d,))

4255   


4256 -    def __str__(self):


4257          return str(self.counter.tolist())

4258   
4259      __repr__ = __str__

4260
```

  


| Home | Trees | Indices | Help | PyDSTool |
| --- | --- | --- | --- | --- |

|  |  |
| --- | --- |
| Generated by Epydoc 3.0.1 on Fri May 4 15:24:14 2012 | http://epydoc.sourceforge.net |
